# Changes in COVID-19-related outcomes, potential risk factors and disparities over time

**DOI:** 10.1017/S0950268821001898

**Published:** 2021-08-10

**Authors:** Youfei Yu, Tian Gu, Thomas S. Valley, Bhramar Mukherjee, Lars G. Fritsche

**Affiliations:** 1 Department of Biostatistics, University of Michigan School of Public Health, Ann Arbor, MI48109, USA; 2 Division of Pulmonary and Critical Care Medicine, Department of Internal Medicine, University of Michigan Medical School, Ann Arbor, MI48109, USA; 3 Institute for Healthcare Policy and Innovation, University of Michigan, Ann Arbor, MI48109, USA; 4 Rogel Cancer Center, University of Michigan Medicine, Ann Arbor, MI48109, USA; 5 Center for Statistical Genetics, University of Michigan School of Public Health, Ann Arbor, MI48109, USA; 6 Department of Epidemiology, University of Michigan School of Public Health, Ann Arbor, MI48109, USA

**Keywords:** Coronavirus, COVID-19, hospitalisation, ICU admission, prognosis, racial disparities, SARS-CoV-2, susceptibility, time-stratified analysis

## Abstract

To investigate temporal trends in coronavirus disease 2019 (COVID-19)-related outcomes and to evaluate whether the impacts of potential risk factors and disparities changed over time, we conducted a retrospective cohort study with 249 075 patients tested or treated for COVID-19 at Michigan Medicine (MM), from 10 March 2020 to 3 May 2021. Among these patients, 26 289 were diagnosed with COVID-19. According to the calendar time in which they first tested positive, the COVID-19-positive cohort were stratified into three-time segments (T1: March–June, 2020; T2: July–December, 2020; T3: January–May, 2021). Potential risk factors that we examined included demographics, residential-level socioeconomic characteristics and preexisting comorbidities. The main outcomes included COVID-19-related hospitalisation and intensive care unit (ICU) admission. The hospitalisation rate for COVID-positive patients decreased from 36.2% in T1 to 14.2% in T3, and the ICU admission rate decreased from 16.9% to 2.9% from T1 to T3. These findings confirm that COVID-19-related hospitalisation and ICU admission rates were decreasing throughout the pandemic from March 2020 to May 2021. Black patients had significantly higher (compared to White patients) hospitalisation rates (19.6% *vs.* 11.0%) and ICU admission rates (6.3% *vs.* 2.8%) in the full COVID-19-positive cohort. A time-stratified analysis showed that racial disparities in hospitalisation rates persisted over time and the estimates of the odds ratios (ORs) stayed above unity in both unadjusted [full cohort: OR = 1.98, 95% confidence interval (CI) (1.79, 2.19); T1: OR = 1.70, 95% CI (1.36, 2.12); T2: OR = 1.40, 95% CI (1.17, 1.68); T3: OR = 1.55, 95% CI (1.29, 1.86)] and adjusted analysis, accounting for differences in demographics, socioeconomic status, and preexisting comorbid conditions (full cohort: OR = 1.45, 95% CI (1.25, 1.68); T1: OR = 1.26, 95% CI (0.90, 1.76); T2: OR = 1.29, 95% CI (1.01, 1.64); T3: OR = 1.29, 95% CI (1.00, 1.67)).

## Introduction

Since the World Health Organization declared coronavirus disease 2019 (COVID-19) a pandemic on 11 March 2020 [1], there have been over 173.1 million confirmed cases worldwide, leading to 3.73 million deaths in the subsequent 15 months [2]. The United States has surpassed 33 million cases, with the state of Michigan reporting 994 935 confirmed cases and over 20 000 deaths as of 5 June 2021 [3]. Studies have found that racial and ethnic minority groups have been disproportionately affected by COVID-19 [4–15]. For example, higher incidence of confirmed cases [5, 6, 8, 12], increased risk of hospitalisation [6, 7, 9, 13], greater fatality burden [5, 8, 10, 11], and higher burden of years of potential life lost [14, 15] were identified for Black individuals when compared to White individuals. Our previous study found certain risk factors, such as specific comorbidities, to affect outcomes in White and Black patients differently [9]. For example, preexisting type 2 diabetes was associated with higher risk of COVID-19-related hospitalisation in White patients but not in Black patients.

Emerging evidence shows an appreciable decline in mortality rates among patients with severe COVID-19 outcomes after the initial wave in March and April 2020 [16–18]. A study conducted in a single health system in New York City found that the hospital mortality rate dropped from 25.6% at the start of the pandemic in March to 7.6% by mid-August 2020, adjusting for demographic and clinical factors [17]. The same trend of improved survival over time was seen among COVID-19 patients requiring hospitalisation [18] or critical care management [16] in England. Racial and ethnic disparities still remain, but recent data showed reduced differences in age-adjusted case fatality rate across ethnic groups [19]. Introduction of vaccines in the beginning of the year 2021 has also impacted COVID outcomes [20, 21]. To understand the dynamics of potential risk factors on COVID-19 outcomes over time, time-stratified analyses are required. Understanding how risk factors changed over the course of the pandemic can enhance the coordination of healthcare resources and help protect the most vulnerable. It can also help us understand whether targeted outreach/prevention efforts in the early months of the pandemic to specific vulnerable subgroups had an effect on improving their outcomes or if disparities persisted over time.

As a follow-up to the smaller cross-sectional study of Gu *et al*. [9], this time-stratified retrospective cohort study aims to examine the sociodemographic and clinical characteristics that are associated with various COVID-19 outcomes by race/ethnicity in a much larger COVID-19 cohort over a 15-month time period, as well as to characterise the risk factor trajectories over time, using electronic health records (EHRs) from Michigan Medicine (MM), a large academic health care system in the State of Michigan.

## Methods

### Study cohort and COVID-19 testing

The study sample consisted of 249 075 patients tested or treated for COVID-19 at MM, the University of Michigan Health System, from 10 March 2020 to 3 May 2021, presenting 26 289 testing positive. The descriptive statistics of the tested cohort are summarised in Table 1. Patients in the tested cohort received one of the five types of diagnostic tests: an in-house polymerase chain reaction (PCR) test (127 615 patients (52.1%)), a point of care PCR test (5781 patients (2.4%)), a commercial PCR test (Viracor; 418 patients (0.2%)), COVID-19 nasopharynx or oropharynx PCR tests deployed by the Michigan Department of Health and Human Services (52 patients (0.02%)), and a small fraction of ribonucleic acid (RNA) tests (three patients (0.001%)); 111 098 tested patients (45.4%) were transferred, tested elsewhere, or had no information on the type of testing they received.

**Table 1.** Descriptive characteristics^a^ of the COVID-19 tested or diagnosed cohort (*n* = 249 075)

	Overall	Negative results	Positive results	Hospitalised	ICU	Deceased	Comparison group
Variable	(*n* = 249 075)	(*n* = 222 786)	(*n* = 26 289)	(*n* = 3071)	(*n* = 844)	(*n* = 485)	(*n* = 26 363)
Age, y							
Mean (s.d.)	44.2 (23.1)	44.5 (23.3)	42.0 (21.6)	53.2 (21.4)	54.6 (20.7)	68.9 (15.4)	43.1 (24.6)
Median (IQR)	45 (40)	46 (39)	41 (37)	57 (33)	59 (26)	71 (22)	43 (42)
<18	32 113 (12.9)	29 462 (13.2)	2651 (10.1)	167 (5.4)	58 (6.9)	2 (0.4)	4925 (18.7)
[18,35)	63 395 (25.5)	54 955 (24.7)	8440 (32.1)	533 (17.4)	97 (11.5)	10 (2.1)	5994 (22.7)
[35,50)	41 045 (16.5)	36 281 (16.3)	4764 (18.1)	473 (15.4)	110 (13)	42 (8.7)	4053 (15.4)
[50,65)	53 950 (21.7)	48 166 (21.6)	5784 (22)	847 (27.6)	274 (32.5)	123 (25.4)	5012 (19)
[65,80)	45 986 (18.5)	42 414 (19)	3572 (13.6)	755 (24.6)	246 (29.1)	168 (34.6)	4565 (17.3)
≥80	12 586 (5.1)	11 508 (5.2)	1078 (4.1)	296 (9.6)	59 (7)	140 (28.9)	1803 (6.8)
Men	108 656 (43.6)	97 094 (43.6)	11 562 (44)	1552 (50.5)	512 (60.7)	296 (61)	12 285 (46.6)
Primary care at MM^b^	98 753 (39.6)	87 921 (39.5)	10 832 (41.2)	1359 (44.3)	294 (34.8)	154 (31.8)	3240 (12.3)
BMI							
Mean (s.d.)	29.0 (7.4)	28.9 (7.3)	29.7 (7.7)	31.5 (8.6)	32.1 (9.9)	30.2 (7.2)	28.4 (7.4)
<18.5	3518 (1.9)	3204 (1.9)	314 (1.6)	48 (1.7)	15 (1.9)	13 (2.9)	314 (2.3)
[18.5,25)	57 716 (30.5)	52 004 (30.8)	5712 (28.4)	536 (18.8)	136 (17.5)	90 (19.9)	4597 (33.2)
[25,30)	58 353 (30.8)	52 480 (31)	5873 (29.2)	844 (29.6)	223 (28.7)	145 (32)	4285 (31)
≥30	69 653 (36.8)	61 408 (36.3)	8245 (40.9)	1424 (49.9)	404 (51.9)	205 (45.3)	4643 (33.6)
Smoking status							
Never	146 795 (65.2)	130 481 (64.7)	16 314 (69.8)	1689 (58.6)	402 (54.8)	163 (41.1)	13 139 (69.1)
Past	58 852 (26.2)	53 026 (26.3)	5826 (24.9)	1055 (36.6)	301 (41.1)	216 (54.4)	3917 (20.6)
Current	19 349 (8.6)	18 122 (9)	1227 (5.3)	140 (4.9)	30 (4.1)	18 (4.5)	1954 (10.3)
Ever	78 201 (34.8)	71 148 (35.3)	7053 (30.2)	1195 (41.4)	331 (45.2)	234 (58.9)	5871 (30.9)
Alcohol consumption	113 508 (68.5)	102 074 (68.8)	11 434 (65.3)	1308 (62)	315 (63.3)	161 (51.9)	7144 (53.7)
Race/ethnicity							
White	182 475 (73.3)	163 976 (73.6)	18 499 (70.4)	2012 (65.5)	512 (60.7)	317 (65.4)	16271 (61.7)
Black	22 524 (9)	19 423 (8.7)	3101 (11.8)	603 (19.6)	193 (22.9)	78 (16.1)	1927 (7.3)
Other^c^	23 770 (9.5)	21 143 (9.5)	2627 (10)	323 (10.5)	76 (9)	38 (7.8)	2444 (9.3)
Unknown^d^	20 306 (8.2)	18 244 (8.2)	2062 (7.8)	133 (4.3)	63 (7.5)	52 (10.7)	5721 (21.7)
NDI, mean (s.d.)	0.1 (0.08)	0.1 (0.08)	0.1 (0.09)	0.13 (0.11)	0.15 (0.12)	0.13 (0.11)	0.11 (0.09)
Population density, persons/mile^2^	2231 (2348.8)	2230.1 (2346.8)	2238.8 (2366.3)	2611.8 (2437.5)	3052.2 (2720.4)	2551.3 (2459.8)	2348.5 (2517)
Comorbidity score, mean (s.d.)	1.9 (1.4)	1.9 (1.4)	1.9 (1.4)	2.9 (1.7)	3.1 (1.6)	3.5 (1.6)	1.2 (1.2)

Abbreviations: BMI, body mass index (calculated as weight in kilograms divided by height in metres squared); COVID-19, coronavirus disease 2019; ICU, intensive care unit; IQR, interquartile range; NDI, 2010 Neighbourhood Socioeconomic Disadvantage Index; MM, Michigan Medicine.

a Unless specified otherwise, numbers shown are numbers (%) of individuals in each category. Percentages are reported as fraction of column totals excluding missing entries.

b At least one visit with a primary care provider at MM within the last 2 years.

c Includes White Hispanic or unknown; Black Hispanic or unknown; Asian Hispanic, non-Hispanic, or unknown; Native American Hispanic, non-Hispanic, or unknown; Pacific Islander Hispanic, non-Hispanic, or unknown; and other Hispanic, non-Hispanic, or unknown.

d Includes missing race and/or ethnicity.

### COVID-19 outcomes

In this study, we primarily focused on three COVID-19-related outcomes among individuals who tested positive: hospitalisation, intensive unit care (ICU) admission, and all-cause mortality. All patients who died post COVID-19 were included in the mortality counts. We performed sensitivity analysis by only considering outcomes that happened within 6 months of a COVID-19 diagnosis. We also investigated the factors associated with being tested (a question related to both access to testing and infection rates) and testing positive for COVID-19 (using a randomly selected untested comparison group described in the Supplementary Material). The corresponding results for these two secondary outcomes are largely reported in the Supplementary Material. The definitions of this ensemble of COVID-19 outcomes are listed in Supplementary Table S1, and the sample sizes for each outcome category are presented in Supplementary Figure S1.

### Definition of demographics, socioeconomic status, comorbidities, and other covariates

We followed the same process described in Gu *et al*. [9] to extract the relevant variables from the EHRs. The sociodemographic variables considered included age, self-reported sex, race/ethnicity, smoking status, alcohol consumption, body mass index (BMI), Neighbourhood Socioeconomic Disadvantage Index (NDI) [22], and population density (in persons per square mile) [22]. NDI is a scale ranging from 0 to 1, with larger values indicating more disadvantaged communities. To characterise the underlying medical conditions of the patients, we considered seven comorbid conditions: respiratory conditions, circulatory conditions, any cancer, type 2 diabetes, kidney diseases, liver diseases and autoimmune diseases, all of which were coded binary. The general health status of patients was represented by a comorbidity score ranging from 0 to 7 that sums up the aforementioned seven conditions. In addition, all patients receiving medical care at least once at any MM primary care location since 1 January 2018 were considered as having MM as their primary care provider.

### Statistical analysis

For each COVID-19-related outcome *Y*_COVID_, we fit a Firth's bias-corrected logistic regression to avoid the potential separation issues in the traditional maximum likelihood estimates. The analysis model is given by1
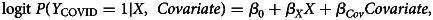
where *X* and *Covariat*e denote the predictors of interest (including *Race*) and the vector of adjustment covariates, respectively. To assess the sensitivity of our analysis to the choice of confounders/adjustment variables, we explored four nested sets of variables for *Covariat*e, respectively labelled adjustment 0–3 (defined in Supplementary Table S1 and results reported in Supplementary Table S2). Our final model (adjustment 3) adjusted for age, sex, race/ethnicity, NDI and the composite comorbidity score. We excluded the cumulative comorbidity score when the factor of interest *X* was one of the individual comorbid conditions. The models for being tested and testing positive further adjusted for population density as that is likely to be related to disease transmission.

We further conducted a time-stratified analysis to assess the changes in racial/ethnic disparities and the effects of other risk factors for severe COVID-19-related outcomes over time since the start of the pandemic. According to the time period in which they first tested positive, the COVID-19-positive cohort were stratified into three groups: Time Period 1 (T1), 10 March 2020–30 June 2020; Time Period 2 (T2), 1 July 2020–31 December 2020; Time Period 3 (T3), 1 January 2021–3 May 2021. Similarly, the COVID-19-negative cohort were stratified based on the time period in which they first received the tests. The logistic regression model (1) was fitted using data for each time period.

The *Race* variable was self-reported and consisted of four categories: non-Hispanic White (White), non-Hispanic Black (Black), other known race/ethnicity, and unknown race/ethnicity. While the disproportionate burden of disease for Hispanics/Latinos is an important public health issue [11, 23], our cohort did not have a sufficiently large sample size to afford the analysis. Therefore, we focus on comparing White and Black patients to evaluate racial/ethnic differences.

To evaluate the possibility of effect modification by race/ethnicity on the effect of potential risk factors, we fit a set of models with interactions by race:
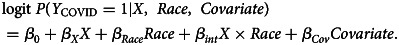


For each model, we report the Firth's bias-corrected estimates of odds ratios along with their associated 95% Wald confidence intervals (CI) and *P* values. For the interaction models, we also obtained the *P* values of the differences in subgroup effects for White and Black patients by testing the null hypothesis *H*_0_: *β*_*int*_ = 0. All analyses were performed using complete cases in R statistical software version 4.0.3 (R Project for Statistical Computing).

## Results

### Descriptive statistics and unadjusted analysis

Table 1 reports the summary statistics for the tested cohort and each of the outcome subgroups. The tested cohort included 249 075 patients, with mean (s.d.) age of 44.2 (23.1), mean (s.d.) BMI of 29.0 (7.4), and 108 656 (43.6%) males. During the study period, more White patients (*n* = 182 475 (73.3%)) were tested than the other races/ethnicities, with 26 829 (73.7%), 112 912 (73.4%), and 39 799 (72.8%) White patients tested in T1, T2, and T3, respectively (Supplementary Table S3). The untested comparison group, which was used only for the secondary outcomes, consisted of 26 363 individuals, with mean (s.d.) age of 43.1 (24.6), mean (s.d.) BMI of 28.4 (7.4), and 12 285 (46.6%) males.

Among the 26 289 patients who tested positive, 3071 (11.7%) were hospitalised, 844 (3.2%) were admitted to the ICU, and 485 (1.8%) died. Older patients, male sex, ever smokers, and increased comorbidity burden tended to be associated with worsened disease severity among COVID-19-positive patients (Table 1). From T1 to T3, the mean and median ages decreased over time for patients who were hospitalised or admitted to the ICU. For example, the mean ages for hospitalised patients were 58.2, 54.2, and 49.0 years in T1, T2, and T3, respectively (Supplementary Table S3). This could potentially be attributed to the increased availability of vaccines for the elderly, the younger population in working-age groups returning to more in-person activities, and a harvesting effect.

Stratifying the descriptive statistics by White and Black patients, we noted differences in covariate distributions between the two groups (Supplementary Table S4). For example, in the tested cohort, Black patients tended to be younger (mean (s.d.) age, 41.4 (21.7) *vs.* 45.7 (23.2)), live in higher populated areas (mean (s.d.) population density in persons per square mile, 3439.4 (2270.7) *vs.* 1985.5 (2201.6)), and be of lower socioeconomic status (mean (s.d.) NDI, 0.21 (0.13) *vs.* 0.08 (0.06)) than White patients. Similar patterns existed in the subgroups (e.g. hospitalised patients) of the tested cohort. We assumed that the data were missing completely at random and used observed complete cases for the analysis. The fractions of missingness for each covariate are presented in Supplementary Table S5.

**Fig. 1.**
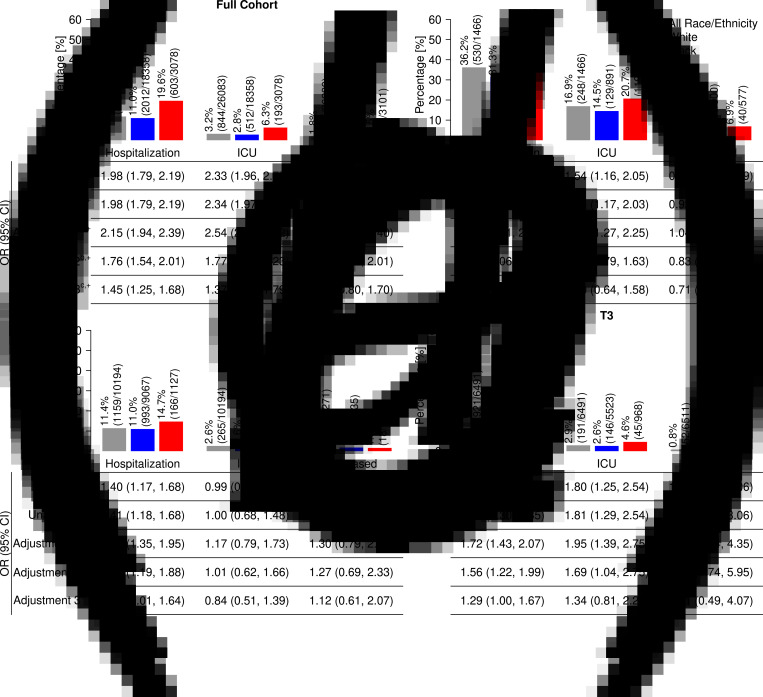
COVID-19 Outcomes Stratified by Race/Ethnicity in Each Time Period. Abbreviations: COVID-19, coronavirus disease 2019; ICU, intensive care unit; OR, odds ratio; T1, 10 March 2020, to 30 June 2020; T2, 1 July 2020, to 31 December 2020; T3, 1 January 2021, to 3 May 2021. ^+^ Logistic regression with Firth's correction. ^a^ Multivariable logistic regression with adjustment 1 (age + sex + race/ethnicity). ^b^ Multivariable logistic regression with adjustment 2 (adjustment 1 + Neighbourhood Socioeconomics Disadvantage Index). ^c^ Multivariable logistic regression with adjustment 3 (adjustment 2 + comorbidity score).

Figure 1 presents the changes in the overall and race-stratified COVID-19 outcomes over time. The overall hospitalisation rate among patients testing positive decreased from 36.2% in T1 to 14.2% in T3, and the overall ICU admission rate decreased from 16.9% to 2.9%. In the sensitivity analysis that only considers outcomes that happened within 6 months of a COVID-19 diagnosis, similar patterns were noted for the hospitalisation and ICU admission rates (Supplementary Figure S2). In the full cohort, Black patients had a significantly higher (unadjusted) hospitalisation rate (603/3078 (19.6%) *vs.* 2012/18 358 (11.0%)), ICU admission rate (193/3078 (6.3%) *vs.* 512/18 358 (2.8%)), and mortality rate (78/3101 (2.5%) *vs.* 317/18 499 (1.7%)) compared to White patients. The unadjusted odds ratios (ORs) for hospitalisation, ICU admission, and death were 1.98 (95% CI (1.79, 2.19)), 2.33 (95% CI (1.96, 2.77)), and 1.48 (95% CI (1.14, 1.91)), respectively. When we examined each time period separately, the point estimates of the hospitalisation rate remained higher for Black patients compared to White patients (Fig. 1) across the study period, but the (unadjusted) ORs were numerically lower in T2 (OR = 1.40, 95% CI (1.17, 1.68)) and T3 (OR = 1.55, 95% CI (1.29, 1.86)) than in T1 (OR = 1.70, 95% CI (1.36, 2.12)). The unadjusted OR for the ICU admission decreased from 1.54 (95% CI (1.16, 2.05)) in T1 to 0.99 (95% CI (0.64, 1.47)) in T2, followed by a rebound in T3 (OR = 1.80, 95% CI (1.25, 2.54)). The mortality rates for Black patients were lower than those of White patients in T1 (40/577 (6.9%) *vs.* 68/900 (7.6%)) and T2 (18/1136 (1.6%) *vs.* 161/9135 (1.8%)), and higher in T3 (11/971 (1.1%) *vs.* 41/5540 (0.7%)), though the differences were not statistically significant due to the small number of patients. At the end of the analysis, 110 patients were still hospitalised (*n* = 62) or still in ICU (*n* = 48).

### Covariate-adjusted analysis using multivariable logistic regression

#### Factors associated with COVID-19 testing

Overall, younger patients, female sex, higher BMI, ever smoker, alcohol consumption, Black race/ethnicity, lower NDI, areas with higher population densities, and accumulation of comorbidities were associated with an increased chance of getting tested (Supplementary Table S6). These associations were fairly consistent over time. For example, Black patients had a higher chance of getting tested across the study period (T1: OR = 1.35, 95% CI (1.26, 1.45); T2: OR = 1.06, 95% CI (0.99, 1.13); T3: OR = 1.27, 95% CI (1.18, 1.36)) than White patients.

#### Factors associated with testing positive

In the full cohort, Black patients had significantly higher risk of testing positive than White patients (OR = 1.48, 95% CI (1.37, 1.61)) (Supplementary Figure S2 and Table S6). Furthermore, age, male sex, ever smoker, and lower NDI were inversely associated with the odds of testing positive. All the comorbidity conditions considered were associated with an increased risk of testing positive. The time-stratified analysis revealed a sharp decrease in the odds of testing positive for Black patients relative to White patients from T1 to T3 (T1: OR = 4.17, 95% CI (3.52, 4.92); T2: OR = 1.19, 95% CI (1.07, 1.31); T3: OR = 1.52, 95% CI (1.36, 1.70)). A complete set of results for testing positive for COVID-19 are reported in the Supplementary Materials.

#### Factors associated with COVID-19-related outcomes among COVID-19-positive patients

Table 2 shows that among the patients who tested positive for COVID-19 through 3 May 2021, Black patients had significantly higher (covariate adjusted) odds of being hospitalised (OR = 1.45, 95% CI (1.25, 1.68)) and admitted to the ICU (OR = 1.37, 95% CI (1.05, 1.79)) compared to White patients. The racial/ethnic differences in mortality (OR = 1.31, 95% CI (0.93, 1.85)) were not statistically significant after covariate adjustment (Supplementary Table S6). The racial/ethnic disparities in hospitalisation rates persisted and the estimates of the ORs stayed above unity over time (T1: OR = 1.26, 95% CI (0.90, 1.76); T2: OR = 1.29, 95% CI (1.01, 1.64); T3: OR = 1.29, 95% CI (1.00, 1.67)). The racial/ethnic disparities in the ICU admission were not significant in each time period (T1: OR = 1.00, 95% CI (0.64, 1.58); T2: OR = 0.84, 95% CI (0.51, 1.39); T3: OR = 1.34, 95% CI (0.81, 2.22)), possibly due to the reduced sample size after stratification.

**Table 2.** Odds ratios (95% confidence intervals)^a^ of COVID-19-related outcomes from logistic regression

Hospitalisation (*Y* = 1) *vs*. not (*Y* = 0)		Full cohort (*n*0 = 23 012, *n*1 = 3071)	Time period 1 (*n*0 = 1169, *n*1 = 636)	Time period 2 (*n*0 = 11 096, *n*1 = 1357)	Time period 3 (*n*0 = 6707, *n*1 = 1073)
**Age (unit: 10-year)**		1.14 (1.12, 1.17)	1.20 (1.11, 1.29)	1.12 (1.08, 1.16)	1.13 (1.08, 1.18)
**Age range, years** REF: [18,35)	[0,18)	0.64 (0.51, 0.81)	2.16 (0.91, 5.12)	0.67 (0.47, 0.94)	0.47 (0.34, 0.66)
	[35,50)	0.86 (0.74, 1.00)	1.18 (0.75, 1.86)	0.71 (0.57, 0.90)	0.81 (0.63, 1.03)
	[50,65)	1.04 (0.90, 1.20)	1.63 (1.07, 2.48)	0.90 (0.73, 1.10)	0.86 (0.68, 1.10)
	[65,80)	1.47 (1.26, 1.71)	2.33 (1.50, 3.61)	1.26 (1.01, 1.56)	1.34 (1.02, 1.75)
	[80,100)	2.37 (1.94, 2.90)	3.71 (2.10, 6.56)	1.97 (1.50, 2.61)	1.94 (1.30, 2.88)
**Male sex**		1.29 (1.17, 1.41)	1.78 (1.38, 2.31)	1.18 (1.03, 1.35)	1.23 (1.05, 1.44)
**BMI**		1.01 (1.00, 1.02)	1.00 (0.99, 1.02)	1.00 (0.99, 1.01)	1.01 (1.00, 1.02)
**BMI range** REF: [18.5,25)	<18.5	1.77 (1.20, 2.59)	3.07 (0.86, 11.0)	2.09 (1.22, 3.59)	1.47 (0.76, 2.84)
	[25,30)	1.11 (0.96, 1.28)	1.31 (0.87, 1.97)	1.09 (0.89, 1.33)	1.04 (0.82, 1.34)
	≥30	1.21 (1.06, 1.38)	1.36 (0.93, 2.00)	1.07 (0.89, 1.29)	1.24 (0.99, 1.55)
**Ever-smoker**		1.16 (1.05, 1.28)	0.99 (0.75, 1.31)	1.24 (1.08, 1.44)	1.13 (0.96, 1.35)
**Smoking status** REF: Never-Smoker	Past-smoker	1.23 (1.11, 1.36)	1.08 (0.80, 1.45)	1.33 (1.14, 1.54)	1.18 (0.99, 1.42)
	Current-smoker	0.85 (0.68, 1.05)	0.65 (0.35, 1.18)	0.76 (0.53, 1.10)	0.97 (0.70, 1.33)
**Alcohol consumption**		0.86 (0.77, 0.95)	0.83 (0.61, 1.12)	0.84 (0.72, 0.98)	0.99 (0.83, 1.19)
**Race/ethnicity** REF: White	Black	1.45 (1.25, 1.68)	1.26 (0.90, 1.76)	1.29 (1.01, 1.64)	1.29 (1.00, 1.67)
	Other / Known Ethnicity^b^	1.28 (1.09, 1.50)	1.23 (0.76, 1.98)	1.35 (1.08, 1.69)	1.20 (0.91, 1.57)
	Other / Unknown Ethnicity	0.50 (0.38, 0.67)	0.66 (0.36, 1.23)	0.45 (0.30, 0.68)	0.42 (0.25, 0.69)
**SES**	NDI	3.62 (2.09, 6.27)	3.61 (0.99, 13.2)	2.26 (0.95, 5.38)	2.68 (1.03, 7.00)
	Population density (1000-people/mi^2^)	1.04 (1.01, 1.06)	1.08 (1.02, 1.14)	1.05 (1.02, 1.08)	1.02 (0.98, 1.06)
**Comorbidity score**		1.49 (1.44, 1.54)	1.38 (1.26, 1.50)	1.60 (1.52, 1.68)	1.55 (1.46, 1.65)
**Comorbidities**^c^	Respiratory	1.47 (1.31, 1.62)	1.30 (0.99, 1.72)	1.75 (1.50, 2.05)	1.40 (1.16, 1.67)
	Circulatory	2.25 (1.99, 2.54)	1.79 (1.31, 2.45)	2.44 (2.04, 2.92)	2.46 (2.00, 3.02)
	Any Cancer	1.77 (1.60, 1.96)	1.55 (1.17, 2.06)	2.02 (1.75, 2.34)	1.82 (1.52, 2.18)
	Type 2 Diabetes	2.06 (1.85, 2.29)	1.90 (1.42, 2.54)	2.26 (1.94, 2.65)	1.91 (1.58, 2.31)
	Kidney	3.97 (3.56, 4.44)	3.53 (2.55, 4.88)	4.46 (3.80, 5.25)	4.55 (3.74, 5.53)
	Liver	2.00 (1.75, 2.30)	1.92 (1.24, 2.97)	2.47 (2.02, 3.02)	1.99 (1.56, 2.53)
	Autoimmune	1.52 (1.36, 1.71)	1.72 (1.27, 2.33)	1.55 (1.31, 1.82)	1.60 (1.31, 1.95)

ICU (*Y* = 1) *vs*. not (*Y* = 0)		Full Cohort (*n*0 = 25 239, *n*1 = 844)	Time Period 1 (*n*0 = 1508, *n*1 = 297)	Time Period 2 (*n*0 = 12 135, *n*1 = 318)	Time Period 3 (*n*0 = 7552, *n*1 = 228)
**Age (unit: 10-year)**		1.18 (1.12, 1.24)	1.23 (1.11, 1.36)	1.16 (1.08, 1.25)	1.03 (0.94, 1.13)
**Age range** REF: [18,35)	[0,18)	1.81 (1.19, 2.76)	2.54 (0.69, 9.33)	1.69 (0.86, 3.34)	1.90 (1.02, 3.55)
	[35,50)	1.21 (0.84, 1.75)	1.40 (0.64, 3.04)	1.03 (0.59, 1.80)	1.05 (0.56, 1.95)
	[50,65)	2.15 (1.57, 2.96)	2.96 (1.47, 5.93)	1.65 (1.03, 2.65)	1.67 (0.95, 2.94)
	[65,80)	3.14 (2.26, 4.36)	3.88 (1.91, 7.88)	2.48 (1.54, 4.00)	2.19 (1.19, 4.04)
	[80,100)	2.77 (1.80, 4.24)	3.27 (1.39, 7.73)	2.25 (1.23, 4.09)	1.06 (0.36, 3.10)
**Male sex**		1.95 (1.63, 2.33)	2.41 (1.69, 3.42)	1.74 (1.32, 2.27)	1.76 (1.27, 2.45)
**BMI**		1.00 (0.99, 1.02)	1.02 (1.00, 1.05)	0.99 (0.97, 1.01)	1.00 (0.98, 1.02)
**BMI range** REF: [18.5,25)	<18.5	3.06 (1.67, 5.60)	1.22 (0.18, 8.47)	5.66 (2.63, 12.2)	2.04 (0.64, 6.49)
	[25,30)	0.91 (0.69, 1.19)	1.15 (0.65, 2.03)	0.91 (0.61, 1.35)	0.68 (0.41, 1.15)
	≥30	1.04 (0.81, 1.33)	1.44 (0.85, 2.45)	0.84 (0.58, 1.23)	0.92 (0.58, 1.44)
**Ever-smoker**		1.24 (1.03, 1.50)	1.09 (0.75, 1.58)	1.40 (1.05, 1.85)	1.21 (0.85, 1.73)
**Smoking status** REF: Never-smoker	Past-smoker	1.35 (1.12, 1.64)	1.22 (0.83, 1.80)	1.49 (1.11, 1.98)	1.31 (0.90, 1.92)
	Current-smoker	0.71 (0.45, 1.13)	0.47 (0.17, 1.30)	0.85 (0.40, 1.81)	0.88 (0.44, 1.76)
**Alcohol consumption**		0.86 (0.70, 1.05)	1.21 (0.80, 1.83)	0.63 (0.46, 0.85)	1.01 (0.67, 1.50)
**Race/Ethnicity** REF: White	Black	1.37 (1.05, 1.79)	1.00 (0.64, 1.58)	0.84 (0.51, 1.39)	1.34 (0.81, 2.22)
	Other / known ethnicity^b^	1.27 (0.94, 1.72)	0.74 (0.35, 1.59)	1.39 (0.90, 2.13)	1.43 (0.85, 2.43)
	Other / unknown ethnicity	0.76 (0.47, 1.22)	0.87 (0.38, 1.97)	0.73 (0.36, 1.47)	0.54 (0.19, 1.58)
**SES**	NDI	8.80 (3.41, 22.7)	6.42 (1.23, 33.5)	7.44 (1.49, 37.1)	2.84 (0.44, 18.4)
	Population density (1000-people/mi^2^)	1.08 (1.04, 1.12)	1.08 (1.00, 1.17)	1.10 (1.04, 1.16)	1.02 (0.95, 1.11)
**Comorbidity score**		1.47 (1.39, 1.56)	1.28 (1.14, 1.44)	1.62 (1.48, 1.77)	1.57 (1.39, 1.76)
**Comorbidities**^c^	Respiratory	1.91 (1.53, 2.38)	1.38 (0.93, 2.05)	2.95 (2.04, 4.26)	1.77 (1.17, 2.68)
	Circulatory	2.84 (2.17, 3.73)	1.56 (0.99, 2.46)	3.88 (2.47, 6.11)	3.88 (2.33, 6.45)
	Any cancer	1.50 (1.24, 1.82)	1.28 (0.88, 1.88)	1.49 (1.11, 1.99)	2.15 (1.48, 3.11)
	Type 2 diabetes	1.90 (1.56, 2.31)	1.57 (1.07, 2.29)	2.48 (1.85, 3.33)	1.43 (0.96, 2.13)
	Kidney	3.94 (3.25, 4.79)	2.83 (1.91, 4.19)	4.67 (3.48, 6.27)	5.04 (3.47, 7.31)
	Liver	1.69 (1.30, 2.18)	1.10 (0.50, 2.01)	2.41 (1.68, 3.45)	1.85 (1.15, 2.97)
	Autoimmune	1.35 (1.08, 1.68)	1.76 (1.18, 2.64)	1.27 (0.90, 1.79)	1.15 (0.74, 1.79)

Abbreviations: BMI, body mass index; ICU, intensive unit care; NDI, Neighbourhood Socioeconomic Disadvantage Index; NA, not applicable.

Odds ratios that are significantly greater than 1 at the level of 0.05 are shaded orange. Time period 1, 10 March 2020, to 30 June 2020; Time period 2, 1 July 2020, to 31 December 2020; Time period 3, 1 January 2021, to 3 May 2021.

a Results were obtained from Firth's multivariable logistic regression model logit *P*(*Y*_COVID_ = 1|*X*, Covariate) = *β*_0_ + *β*_X_*X* + *β*_Cov_Covariate, where *Y*_COVID_ is the COVID-19 outcomes (i.e. positive test results, hospitalisation, admission to ICU, or mortality). Covariate = age + sex + race + NDI + comorbidity score.

b Includes White Hispanic or unknown; Black Hispanic or unknown; Asian Hispanic, non-Hispanic, or unknown; Native American Hispanic, non-Hispanic, or unknown; Pacific Islander Hispanic, non-Hispanic, or unknown; and other Hispanic, non-Hispanic, or unknown.

c Not adjusted for composite comorbidity score.

Additionally, age, male sex, smoking, living in densely populated areas or disadvantaged neighbourhoods, and heavier overall comorbidity burden were positively associated with hospitalisation and ICU admission in the full COVID-positive cohort (Table 2). Most of the associations in each time period stayed in the same direction as in the full cohort analysis (Table 2). Specifically, older patients (every 10-year increase in age) were consistently associated with increased risk of being hospitalised (T1: OR = 1.20, 95% CI (1.11, 1.29); T2: OR = 1.12, 95% CI (1.08, 1.16); T3: OR = 1.13, 95% CI (1.08, 1.18)). Among the underlying conditions, kidney diseases in general had the largest ORs of hospitalisation (T1: OR = 3.53, 95% CI (2.55, 4.88); T2: OR = 4.46, 95% CI (3.80, 5.25); T3: OR = 4.55, 95% CI (3.74, 5.53)) and ICU admission (T1: OR = 2.83, 95% CI (1.91, 4.19); T2: OR = 4.67, 95% CI (3.48, 6.27); T3: OR = 5.04, 95% CI (3.47, 7.31)) across the study periods.

#### Interaction analysis by race/ethnicity within the COVID-19-positive cohort

**Fig. 2.**
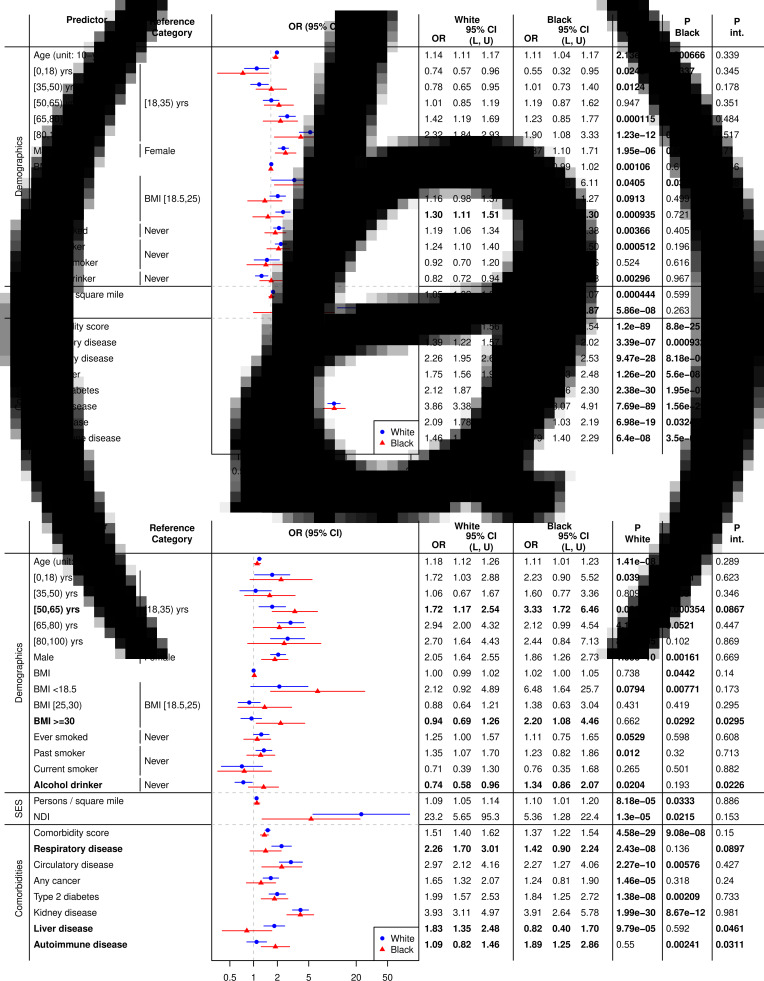
Hospitalisation (A) and ICU Admission (B) for Black and White Patients in the Full Cohort. Abbreviations: BMI, body mass index; NDI, Neighbourhood Socioeconomic Disadvantage Index. The results were from the model *logit P*(*Y*_*COVID*_ = 1|*X*, *Covariate*) = *β*_0_ + *β*_*X*_*X* + *β*_*Race*_*Race* + *β*_*int*_*X* × *Race* + *β*_*Cov*_*Covariate*, where *Y*_*COVID*_ denotes hospitalisation (A) or ICU admission (B), and *Covariate* = age + sex + NDI ( + comorbidity score in the demographic and socioeconomic status models). Results that are statistically significant at the level of 0.05 are in bold.

Figure 2 displays the ORs of potential risk factors stratified by race/ethnicity for the hospitalisation outcome (Fig. 2A) and ICU admission (Fig. 2B). The main effects of the seven comorbid conditions considered were significant in both Black and White patients for hospitalisation, while none of the comorbidities exhibited significant interaction effects. For example, the OR of kidney diseases for Black and White patients were 3.88 (95% CI (3.07, 4.91), *P* < 0.001) and 3.86 (95% CI (3.38, 4.40), *P* < 0.001), respectively, and the interaction *P* value was 0.96. Higher NDI (i.e. lower socioeconomic status) had significant positive associations with hospitalisation in White patients (OR = 9.29, 95% CI (4.15, 20.8), *P* < 0.001), but not in Black patients (OR = 1.64, 95% CI (0.69, 3.87), *P* = 0.263), with the interaction being significant (*P_int_* = 0.004). For the ICU admission (Fig. 2B), significant interaction effects were observed in patients with obesity (BMI ≥30) (White: OR = 0.94, 95% CI (0.69, 1.26), *P* = 0.662; Black: OR = 2.20, 95% CI (1.08, 4.46), *P* = 0.029; *P_int_* = 0.030), alcohol drinkers (White: OR = 0.74, 95% CI (0.58, 0.96), *P* = 0.020; Black: OR = 1.34, 95% CI (0.86, 2.07), *P* = 0.193; *P_int_* = 0.023), and patients with underlying liver diseases (White: OR = 1.83, 95% CI (1.35, 2.48), *P* < 0.001; Black: OR = 0.82, 95% CI (0.40, 1.70), *P* = .592; *P_int_* = 0.046) and autoimmune diseases (White: OR = 1.09, 95% CI (0.82, 1.46), *P* = 0.550; Black: OR = 1.89, 95% CI (1.25, 2.86), *P* = 0.002; *P_int_* = 0.031).

## Discussion

In this study, we evaluated the potential risk factors and racial/ethnic disparities for COVID-19 prognosis using a larger cohort from the same healthcare system that covers a longer time frame than Gu *et al*. [9]. We observed that Black patients and patients with higher comorbidity burden were more susceptible to COVID-19 and more likely to be hospitalised, a result consistent with previous findings in the literature. On the other hand, the association between age and positive test results reversed compared to what was reported in Gu *et al*. [9], such that younger people, especially those aged 18–35 years, were more likely to be tested positive. The finding aligns with recent data from the Centers for Disease Control and Prevention, which shows an increase in the COVID-19 incidence in persons aged <30 years and a decrease in the median age for confirmed cases in May−August 2020 [24].

A fraction of patients who were hospitalised (*n* = 249 of 3071 (8.1%)) in our sample were transferred from external hospitals, mainly from the Detroit Metro area. These transferred patients tended to have more severe symptoms and different distributions in sociodemographic characteristics than non-transferred patients. It is important to note the temporal changes in the context of care at MM (Supplementary Table S7). There was an enrichment in transferred patients in T1 (*n* = 123 (19.3%)) compared to T2 (*n* = 76 (5.6%)) and T3 (*n* = 50 (4.7%)) for those hospitalised. However, a sensitivity analysis that excluded patients who did not receive their primary care at MM was consistent with the overall analysis, namely improved outcomes and reduced but persisting health disparities over time (Supplementary Table S8).

Our definition of COVID-19 related hospitalisation, ICU admission, and mortality may be liberal. We performed a sensitivity analysis restricting the time period to six months since the first positive diagnosis. For example, of the 485 deaths, 458 (94.4%) happened within six months. The results of the sensitivity analysis (Supplementary Figure S3) showed consistent patterns with the analysis based on unfiltered outcomes (Fig. 1) with respect to the changes in the rates and ORs of the three outcomes. Specifically, the overall hospitalisation rate, ICU admission rate, and mortality rate decreased from 34.2%, 16.5%, and 7.1% to 9.6%, 1.9%, and 0.8%, respectively.

Our findings suggest that the COVID-19-related hospitalisation, ICU admission, and mortality rates declined from T1 to T2, which may be partly explained by the rapid increase in testing and adoption of preventive measures. The rates of these severe outcomes remained relatively steady from T2 to T3. However, racial/ethnic disparities persisted over time. This study also confirms a number of potential risk factors for COVID-19-related outcomes: older age, male sex, Black race/ethnicity, preexisting conditions, and lower socioeconomic status. Among the examined comorbidities, underlying kidney diseases in general had the largest odds ratios for the COVID-19-related outcomes. The interaction analysis identified several risk factors that affected the hospitalisation and ICU admission differently in Black and White patients. Specifically, underlying respiratory diseases and liver diseases increased the risk of hospitalisation in the subgroup of White patients, while the effects on Black patients were not significant. On the other hand, obesity and autoimmune diseases were positively associated with ICU admission in Black patients but not in White patients.

There are several limitations of our study. First, the sample cannot be considered a population-based random sample, as MM prioritised testing to those presenting COVID-19 symptoms or at greatest risk of exposure, particularly during the early stages of the pandemic when the availability of tests was limited. Second, since the hospitalisation records were available only for those treated at MM, not all hospitalised patients were captured by our data. Patients hospitalised elsewhere may be classified as non-hospitalised, which may lead to biased estimates. Finally, 486 out of the 52 325 people who were first tested in T3 received at least one vaccination dose by May 3, but we did not consider vaccination as a covariate, which may explain part of the reduction in hospitalisation and mortality. Despite the limitations, our study assessed the temporal trend in COVID-19-related outcomes and evaluated whether the impacts of potential risk factors and disparities changed over time. Elderly adults, Black patients, and patients with underlying health conditions were disproportionately affected by COVID-19 over the course of the pandemic, which calls for vaccine prioritisation and targeted outreach in these population groups.

## Supplementary material

For supplementary material accompanying this paper visit http://dx.doi.org/10.1017/S0950268821001898. click here to view supplementary material

## Data

The individual level data cannot be shared publicly due to patient confidentiality. The data underlying the results presented in the study are available from University of Michigan Data Office for Clinical & Translational Research for researchers who meet the criteria for access to confidential data.
