# Supplementary material for: Changes in COVID-19-related outcomes, potential risk factors and disparities over time
Source: Epidemiol Infect. 2021 Aug 10;149:e192. doi: 10.1017/S0950268821001898 (PMC8376857; doi:10.1017/S0950268821001898)
Supplement: Supplementary file 1 [file S0950268821001898sup001.docx]

*Epidemiology and Infection*

**Changes in COVID-19-related outcomes, potential risk factors and disparities over time**

Youfei Yu, Tian Gu, Thomas S. Valley, Bhramar Mukherjee, Lars G. Fritsche

**Supplementary Material**

**Supplementary Methods.** Analysis of the Susceptibility of COVID-19.

**Supplementary Results.** Factors associated with the Susceptibility of COVID-19.

**Supplementary Figure S1.** Flow Diagram of Patients Tested and Subsequent Patient Outcomes, Stratified by Race/Ethnicity.

**Supplementary Figure S2.** COVID-19 Testing and Susceptibility Stratified by Race/Ethnicity in Each Time Period.

**Supplementary Figure S3.** COVID-19 Outcomes that Occurred within Six Months of a COVID-19 Diagnosis Stratified by Race/Ethnicity in Each Time Period

**Supplementary Figure S4.** COVID-19 Susceptibility for White and Black Patients in the Full Cohort.

**Supplementary Table S1.** Sources of All Variables and Relevant Definitions

**Supplementary Table S2.** Odds Ratios of COVID-19 Outcomes From Logistic Regression for the Full Cohort.

**Supplementary Table S3.** Characteristics of the COVID-19 Tested or Diagnosed Cohort, Stratified by Time Periods.

**Supplementary Table S4.** Descriptive Characteristics of the COVID-19 Tested or Diagnosed Cohort Stratified by White and Black Patients.

**Supplementary Table S5.** Missingness of the Variables in the Full Cohort, White, and Black Patients.

**Supplementary Table S6.** Odds Ratios of COVID-19 Outcomes from Logistic Regression, Stratified by Time Periods.

**Supplementary Table S7.** Proportions of Transferred Patients by Outcome and Time Periods.

**Supplementary Table S8.** Sensitivity Analysis Using Patients with Primary Care at Michigan Medicine, Stratified by Time Periods.

**Supplementary Methods. Analysis of the Susceptibility of COVID-19**

*Selection of the Comparison Group*

In order to account for selection bias in our sample, we created a comparative group of untested individuals from the MM database. The comparative group included 26363 randomly selected individuals who were alive on 3 May 2020 and have had at least one encounter with MM since 23 April 2020. The detailed inclusion and exclusion criteria are presented in Supplementary Table S1.

*Statistical Methods*

We examined the characteristics associated with testing positive using the untested cohort described in the previous section for comparison. The analysis model is the same as Model (1) in the main text,

$$\begin{aligned} \mathrm{logit} P\left( \left. Y_{\mathrm{COVID}}=1 \right|X, Covariate \right)=\beta_{0}+\beta_{X}X+\beta_{Cov}Covariate, \end{aligned}$$

where $X$ and $Covariate$ denote the risk factor of interest and the vector of covariates, respectively. Results are summarized in the Supplementary Materials (Supplementary Figures S2 and S3, and Supplementary Table S6).

**Supplementary Results. Factors associated with the Susceptibility of COVID-19.**

Supplementary Figure S2 indicated the differences in COVID-19 susceptibility between White and Black patients. In the full cohort, the Black patients had significantly higher test positivity rate (3101/22524 [13.8%]) than White patients (18499/182475 [10.1%]). When the cohort were stratified by time periods, the test positivity rate remained high for Black patients compared to White patients, and the (unadjusted) odds ratios (ORs) were lower in Q2 and Q3 (Q2: OR=1.16, 95% CI [1.09, 1.24]; Q3: OR=1.33, 95% CI [1.23, 1.43]) than Q1 (OR=4.42, 95% CI [3.95, 4.74]).

In the full cohort, several factors were identified to be statistically different between the COVID-19-positive group and the untested controls. Specifically, Black patients had significant higher risk of testing positive than White patients (OR=1.48, 95% CI [1.37, 1.61]) (Supplementary Table S6). Every 10-year increase in age was inversely associated with the odds of testing positive (OR=0.92, 95% CI [0.91, 0.93]), as was male sex (OR=0.85, 95% CI [0.81, 0.88]) and ever smokers (OR=0.89, 95% CI [0.84, 0.94]). All of the comorbidity conditions considered were associated with an increased risk of having positive test results, with odds ratios ranging from 1.25 (95% CI [1.18, 1.32]) for any cancer to 2.92 (95% CI [2.62, 3.26]) for liver diseases. The stratified analyses showed that most of the associations in each time period stayed in the same direction as in the full cohort analysis (Supplementary Table S6).

Significant associations with positive test results were identified for overall comorbidity burden in both White (OR=1.61, 95% CI [1.58, 1.65], P<.001) and Black patients (OR=1.75, 95% CI [1.66, 1.85], P<.001), but it posed a higher risk on Black patients (P*_int_*=.004). Circulatory diseases, any cancer, type 2 diabetes, kidney diseases, and autoimmune diseases showed similar directional results (Supplementary Figure S4).


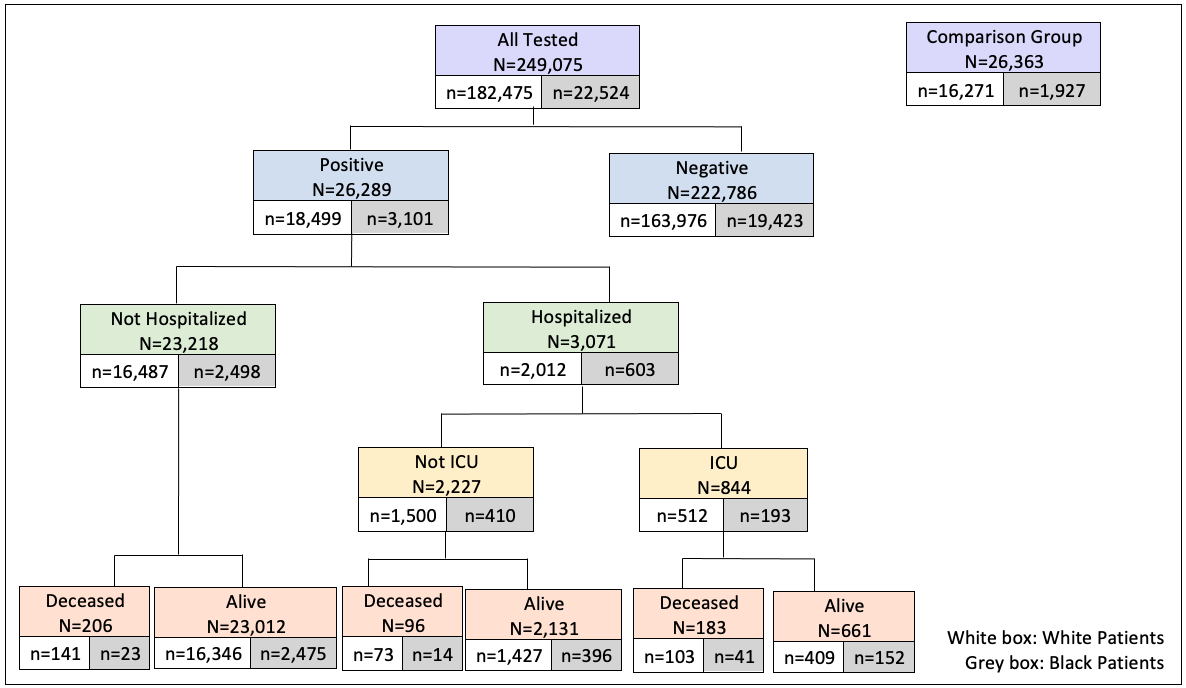


**Supplementary Figure S1. Flow Diagram of Patients Tested and Subsequent Patient Outcomes, Stratified by Race/Ethnicity**

For each COVID-19 outcome, we listed the total number of patients, as well as the total number of White patients (in the white box) and Black patients (in the grey box).

**Supplementary Figure S2. COVID-19 Testing and Susceptibility Stratified by Race/Ethnicity in Each Time Period.**

Abbreviations: COVID-19, coronavirus disease 2019; ICU, intensive care unit; OR, odds ratio; T1, 10 March 2020, to 30 June 2020; T2, 1 July 2020, to 31 December 2020; T3, 1 January 2021, to 3 May 2021. For the outcome of getting tested and testing positive, patients were classified into each time period based on the earliest test time of getting tested and earliest time of testing positive, respectively.

^+^ Logistic regression with Firth’s correction.

^a^ Multivariable logistic regression with adjustment 1 (age + sex + race/ethnicity). Population density was also adjusted in the susceptibility (i.e., having positive test results) models.

^b^ Multivariable logistic regression with adjustment 2 (adjustment 1 + Neighborhood Socioeconomics Disadvantage Index).

^c^ Multivariable logistic regression with adjustment 3 (adjustment 2 + comorbidity score).

**Supplementary Figure S3. COVID-19 Outcomes that Occurred within Six Months of a COVID-19 Diagnosis Stratified by Race/Ethnicity in Each Time Period**

Abbreviations: COVID-19, coronavirus disease 2019; ICU, intensive care unit; OR, odds ratio; T1, 10 March 2020, to 30 June 2020; T2, 1 July 2020, to 31 December 2020; T3, 1 January 2021, to 3 May 2021. For the outcome of getting tested and testing positive, patients were classified into each time period based on the earliest test time of getting tested and earliest time of testing positive, respectively.

^+^ Logistic regression with Firth’s correction.

^a^ Multivariable logistic regression with adjustment 1 (age + sex + race/ethnicity). Population density was also adjusted in the susceptibility (i.e., having positive test results) models.

^b^ Multivariable logistic regression with adjustment 2 (adjustment 1 + Neighborhood Socioeconomics Disadvantage Index).

^c^ Multivariable logistic regression with adjustment 3 (adjustment 2 + comorbidity score).

**Supplementary Figure S4. COVID-19 Susceptibility for White and Black Patients in the Full Cohort.**

Abbreviations: BMI, body mass index; NDI, Neighborhood Socioeconomic Disadvantage Index.

The results were from model $logit P\left( \left. Y_{COVID}=1 \right|X, Covariate \right)=\beta_{0}+\beta_{X}X+\beta_{Race}Race+\beta_{int}X\times Race+\beta_{Cov}Covariate$, where $Y_{COVID}$ denotes hospitalization (A) or ICU admission (B), and $Covariate$=age + sex + NDI + population density (+ comorbidity score in the demographic and socioeconomic status models). Results that are statistically significant at the level of 0.05 are bolded.

**Supplementary Table S1. Sources of All Variables and Relevant Definitions**

| **Variable** | **Definition** | **Sources** |
| --- | --- | --- |
| Age | Age of patient as of the data pull: 3 May 2020 | Electronic Health Record (EPIC) |
| Male | Gender of patient as reported. | Electronic Health Record (EPIC) |
| Primary Care at MM | If the patient has had an encounter in any of the primary care locations at MM since 1 January 2018, then 1; otherwise 0 | Derived from the Electronic Health Record |
| BMI | Excluded entries if (1) age at BMI measurement was missing or below 18 years, (2) height and/or weight were missing, (3) height measurements were below 69 cm or above 234 cm, (4) weight was above 400 kg, (5) BMI deviated more than one unit from BMI calculated from height and weight (BMI = weight [in kg] / height [in m]^2 ). Outliers for multiple values per person were defined as values that exceeded the median BMI +/- 3 x the median absolute deviation (MAD). Final BMI values was calculated as the median BMI of the remaining entries. | Derived from the Electronic Health Record |
| Ever-Smoker | If the last reported smoking status is "never", but reported smoking before 1; If the last reported smoking status is "never", and never reported smoking before, then 0 | Derived from the Electronic Health Record |
| **Smoking Status** |  | Self-Reported under Patient History in EHR |
| Never | If the patient never say that they are a 'former' smoker or 'current' smoker then 1, otherwise 0 |  |
| Past | If the last smoking status is "former", then 1 |  |
| Current | If the last smoking status is "current", then 1 |  |
| **Alcohol Consumption** | If the reported alcohol drinker status in the EHR was reported "yes" at least once; and never reported alcohol drinker before, then 0 | Self-Reported under Patient History in EHR |
| **Race/Ethnicity** |  | Patient Reported - Derived from the Electronic Health Record |
| White | If race was reported as "Caucasian" and ethnicity as "Hispanic or Latino" |  |
| Black | If race was reported as "African American" and ethnicity as "Hispanic or Latino" |  |
| Other / Known Ethnicity | If race was not reported as "African American" or "Caucasian"' and ethnicity was reported as "Non-Hispanic or Latino" or "Hispanic or Latino" |  |
| Other / Unknown Ethnicity | If race and/or ethnicity were missing |  |
| **SES** | Data defined by US census tract (based on residential address available in each patient’s EHR) for the year 2010 from the US Census and the American Community Survey (ACS). | The boundaries for the census tracts were normalized by 2010 tract boundaries using the Longitudinal Tract Data Base (Logan, Xu, and Stults, 2014). |
| NDI | 2010 Neighborhood Socioeconomic Disadvantage Index (with Proportion Black): mean of proportion of Population in Poverty; Unemployed; with Public Assistance Income; and Female-Headed Families with children. |  |
| Population density^a^ (1000-people/mi^2^) | Population density of the neighborhood that the patient lives in. |  |
| **Comorbidities** |  | Electronic Health Record (EPIC) |
| Respiratory Diseases | At least one of the following observed phecodes and their subcodes:  464, 465, 465.2, 465.4, 470, 471, 472, 473, 473.1, 473.3, 473.4, 474, 474.1, 474.2, 475, 475.9, 476, 477, 478, 479, 480, 480.1, 480.11, 480.12, 480.13, 480.2, 480.3, 480.5, 481, 483, 495, 495.1, 495.11, 495.2, 496, 496.1, 496.2, 496.21, 496.3, 497, 498, 499, 500, 500.1, 500.2, 501, 502, 503, 504, 504.1, 505, 506, 507, 508, 509, 509.1, 509.2, 509.3, 509.5, 509.8, 510, 510.2, 512, 512.1, 512.2, 512.3, 512.7, 512.8, 512.9, 513, 513.3, 513.31, 513.32, 513.4, 513.8, 514, 514.1, 514.2, 516, 516.1, 519, 519.1, 519.2, 519.8, 519.9 |  |
| Circulatory Diseases | At least one of the following observed phecodes and their subcodes:  394, 394.1, 394.2, 394.3, 394.4, 394.7, 395, 395.1, 395.2, 395.3, 395.4, 395.6, 396, 401, 401.1, 401.2, 401.21, 401.22, 401.3, 411, 411.1, 411.2, 411.3, 411.4, 411.41, 411.8, 411.9, 414, 414.2, 415, 415.1, 415.11, 415.2, 415.21, 416, 418, 418.1, 420, 420.1, 420.2, 420.21, 420.22, 420.3, 425, 425.1, 425.11, 425.12, 425.2, 425.8, 426, 426.2, 426.21, 426.22, 426.23, 426.24, 426.25, 426.3, 426.31, 426.32, 426.4, 426.7, 426.8, 426.9, 426.91, 426.92, 427, 427.1, 427.11, 427.12, 427.2, 427.21, 427.22, 427.3, 427.4, 427.41, 427.42, 427.5, 427.6, 427.61, 427.7, 427.8, 427.9, 428, 428.1, 428.2, 428.3, 428.4, 429, 429.1, 429.2, 429.3, 429.9, 430, 430.1, 430.2, 430.3, 433, 433.1, 433.11, 433.12, 433.2, 433.21, 433.3, 433.31, 433.32, 433.5, 433.6, 433.8, 440, 440.1, 440.2, 440.21, 440.22, 440.9, 441, 441.1, 441.2, 442, 442.1, 442.11, 442.2, 442.3, 442.4, 442.8, 443, 443.1, 443.7, 443.8, 443.9, 444, 444.1, 444.2, 444.5, 446, 446.1, 446.2, 446.3, 446.4, 446.5, 446.6, 446.7, 446.8, 446.9, 447, 447.1, 447.7, 448, 450, 451, 451.2, 452, 452.1, 452.2, 452.8, 453, 454, 454.1, 454.11, 455, 456, 457, 457.2, 457.3, 458, 458.1, 458.2, 458.9, 459, 459.1, 459.7, 459.9 |  |
| Any Cancer | At least one of the following observed phecodes and their subcodes:  145, 145.2, 145.3, 145.4, 149, 149.1, 149.2, 149.3, 149.4, 149.5, 149.9, 150, 151, 153, 153.2, 153.3, 155, 155.1, 157, 158, 159, 159.2, 159.3, 159.4, 164, 165, 165.1, 170, 170.1, 170.2, 172, 172.1, 172.11, 172.2, 172.21, 172.22, 172.3, 174, 174.1, 174.11, 175, 180, 180.1, 180.3, 182, 184, 184.1, 184.11, 184.2, 185, 187, 187.1, 187.2, 189, 189.1, 189.11, 189.12, 189.2, 189.21, 189.4, 190, 191, 191.1, 191.11, 193, 194, 195, 195.1, 195.3, 196, 197, 198, 198.1, 198.2, 198.3, 198.4, 198.5, 198.6, 198.7, 199.4, 200, 200.1, 201, 202, 202.2, 202.21, 202.22, 202.23, 202.24, 204, 204.1, 204.11, 204.12, 204.2, 204.21, 204.22, 204.3, 204.4, 209 |  |
| Type 2 Diabetes | At least one of the following observed phecode and their subcodes: 250.2 |  |
| Kidney Diseases | At least one of the following observed phecodes and their subcodes: 585 |  |
| Liver Diseases | At least one of the following observed phecodes and their subcodes: 571 |  |
| Autoimmune Diseases | At least one of the following observed phecodes and their subcodes: 242.1, 250.1, 335, 557.1, 694.1, 695.4, 696.4, 697, 704.1, 714.1, 717 |  |
| Comorbidity Score | The summation of 7 comorbidities values above, ranging from 0 to 7 |  |
| **COVID-19 Outcomes** |  | Derived from RDW's COVID Registry |
| COVID-19 Tested | Patients who were tested for COVID-19 at the time of data pull. |  |
| COVID-19 Positive | Patients who tested positive at least once for COVID-19 |  |
| COVID-19 Negative | Patients who always tested negative for COVID-19 |  |
| Non-Hospitalized | Patients in the positive COVID cohort who have no inpatient stays after 5 March 2020 |  |
| Hospitalized | Patients in the positive COVID cohort who checked in as an inpatient after 5 March 2020 at least once. |  |
| ICU | Patients in the positive COVID cohort who checked into the ICU during their inpatient stay after 5 March 2020. |  |
| Deceased | Patients in the cohort who have died based on their Electronic Health Record. | Electronic Health Record (EPIC) |
| **Comparison Group** | Randomly picked cohort of patients who are not part of the COVID-19 cohort (Tested, Positive, Negative), who are alive and who have had an encounter in MM (Inpatient, Outpatient or Emergency) since 2012-04-23. We created an untested comparison group (n=26363) from the MM database, which is a similar-sized random sample of contemporaneous patients. Specifically, we initially extracted 30,000 individuals before limiting the group to patients who (1) were alive at the time of data pull, (2) have had encounters after 2012-04-22 and (3) who had inpatient, outpatient and/or emergency visits. At the time of the last update for COVID-19 outcomes (2 September 2020), all patients in the comparison group were alive. | Derived from the Electronic Health Record |
| **Adjustments** |  |  |
| Adjustment 0 | Unadjusted |  |
| Adjustment 1 | age + sex + race/ethnicity (+ population density)* |  |
| Adjustment 2 | adjustment 1 + NDI |  |
| Adjustment 3 | adjustment 2 + comorbidity score |  |
| **Time Period** |  |  |
| Time Period 1 | 10 March 2020, to 30 June 2020 |  |
| Time Period 2 | 1 July 2020, to 31 December 2020 |  |
| Time Period 3 | 1 January 2021, to 3 May 2021 |  |

Abbreviations: MM, Michigan Medicine; ICU, intensive care unit; BMI, body mass index; SES, social economics status; NDI, 2010 Neighborhood Socioeconomic Disadvantage Index.

^a^ The population density is used only in the tested positive/susceptibility model as a covariate.

**Supplementary Table S2.** Odds Ratios of COVID-19 Outcomes From Logistic Regression for the Full Cohort

| **Positive (1) vs Comparison Group (0)** | | **Unadjusted**  **(n_0_=26353 n_1_=26289)** | **Adjustment 1**  **(n_0_=21441, n_1_=20727)** | **Adjustment 2**  **(n_0_=21440, n_1_=20727)** | **Adjustment 3**  **(n_0_=20410, n_1_=19723)** |
| --- | --- | --- | --- | --- | --- |
| **Variable** | | **OR (95% CI)** |  |  |  |
| **Age (unit: 10-year)** | | 0.98 (0.97, 0.99) | 1.04 (1.04, 1.05) | 1.04 (1.04, 1.05) | 0.92 (0.91, 0.93) |
| **Age Range**  REF: [18,35) | [0,18) | 0.38 (0.36, 0.41) | 0.42 (0.39, 0.45) | 0.42 (0.39, 0.45) | 0.48 (0.45, 0.52) |
|  | [35,50) | 0.84 (0.79, 0.88) | 1.06 (1.00, 1.13) | 1.07 (1.01, 1.14) | 0.87 (0.81, 0.93) |
|  | [50,65) | 0.82 (0.78, 0.86) | 1.08 (1.02, 1.15) | 1.09 (1.03, 1.15) | 0.68 (0.64, 0.73) |
|  | [65,80) | 0.56 (0.53, 0.59) | 0.76 (0.71, 0.81) | 0.77 (0.72, 0.82) | 0.39 (0.36, 0.42) |
|  | [80,100) | 0.43 (0.39, 0.46) | 0.62 (0.57, 0.69) | 0.63 (0.57, 0.69) | 0.28 (0.25, 0.31) |
| **Male Sex** | | 0.90 (0.87, 0.93) | 0.86 (0.83, 0.90) | 0.86 (0.83, 0.89) | 0.85 (0.81, 0.88) |
| **BMI** | | 1.02 (1.02, 1.03) | 1.03 (1.02, 1.03) | 1.03 (1.03, 1.03) | 1.02 (1.02, 1.02) |
| **BMI Range**  REF: [18.5,25) | <18.5 | 0.81 (0.69, 0.95) | 0.83 (0.69, 0.99) | 0.84 (0.70, 1.00) | 0.79 (0.66, 0.96) |
|  | [25,30) | 1.10 (1.04, 1.17) | 1.26 (1.19, 1.35) | 1.27 (1.19, 1.35) | 1.25 (1.17, 1.33) |
|  | $\boldsymbol{\geq}$30 | 1.43 (1.36, 1.51) | 1.64 (1.54, 1.74) | 1.66 (1.56, 1.76) | 1.44 (1.35, 1.53) |
| **Ever-Smoker** | | 0.97 (0.93, 1.01) | 0.95 (0.90, 1.00) | 0.96 (0.91, 1.01) | 0.89 (0.84, 0.94) |
| **Smoking Status**  REF: Never-Smoker | Past-Smoker | 1.20 (1.14, 1.25) | 1.20 (1.13, 1.27) | 1.08 (1.02, 1.15) | 1.08 (1.02, 1.15) |
|  | Current-Smoker | 0.51 (0.47, 0.55) | 0.50 (0.46, 0.55) | 0.52 (0.48, 0.57) | 0.52 (0.48, 0.57) |
| **Alcohol Consumption** | | 1.62 (1.55, 1.70) | 1.59 (1.51, 1.67) | 1.58 (1.50, 1.66) | 1.72 (1.63, 1.82) |
| **Race/Ethnicity**  REF: White | Black | 1.42 (1.33, 1.50) | 1.51 (1.41, 1.61) | 1.68 (1.56, 1.81) | 1.48 (1.37, 1.61) |
|  | Other / Known Ethnicity | 0.95 (0.89, 1.00) | 0.96 (0.89, 1.02) | 0.97 (0.90, 1.03) | 0.99 (0.92, 1.06) |
|  | Other / Unknown Ethnicity | 0.32 (0.30, 0.34) | 0.27 (0.25, 0.29) | 0.28 (0.26, 0.30) | 0.38 (0.35, 0.41) |
| **SES** | Population density (1000-people/mi^2^) | 0.98 (0.97, 0.99) | 0.98 (0.97, 0.98) | 0.99 (0.98, 0.99) | 0.99 (0.98, 1.00) |
|  | NDI | 0.65 (0.53, 0.81) | 0.40 (0.31, 0.52) | 0.40 (0.31, 0.52) | 0.28 (0.21, 0.37) |
| **Comorbidity Score** | | 1.57 (1.54, 1.59) | 1.66 (1.63, 1.70) | 1.67 (1.64, 1.70) | 1.67 (1.64, 1.70) |
| **Comorbidities** | Respiratory | 3.08 (2.97, 3.20) | 2.90 (2.78, 3.03) | 2.89 (2.77, 3.01) | NA |
|  | Circulatory | 2.33 (2.24, 2.41) | 2.49 (2.38, 2.60) | 2.50 (2.39, 2.61) | NA |
|  | Any Cancer | 1.19 (1.14, 1.24) | 1.25 (1.18, 1.32) | 1.25 (1.18, 1.32) | NA |
|  | Type 2 Diabetes | 2.08 (1.96, 2.21) | 1.98 (1.85, 2.12) | 2.02 (1.88, 2.16) | NA |
|  | Kidney | 2.83 (2.62, 3.05) | 2.58 (2.37, 2.81) | 2.62 (2.41, 2.85) | NA |
|  | Liver | 3.14 (2.84, 3.47) | 2.89 (2.59, 3.23) | 2.92 (2.62, 3.26) | NA |
|  | Autoimmune | 2.32 (2.18, 2.47) | 2.16 (2.01, 2.31) | 2.16 (2.02, 2.32) | NA |
| **Hospitalization (1) vs Not (0)** | | **Unadjusted**  **(n_0_=23012, n_1_=3071)** | **Adjustment 1**  **(n_0_=23010, n_1_=3071)** | **Adjustment 2**  **(n_0_=18109, n_1_=2452)** | **Adjustment 3**  **(n_0_=17360, n_1_=2204)** |
| **Age (unit: 10-year)** | | 1.33 (1.31, 1.35) | 1.34 (1.32, 1.37) | 1.32 (1.29, 1.35) | 1.14 (1.12, 1.17) |
| **Age Range**  REF: [18,35) | [0,18) | 1.00 (0.84, 1.20) | 0.95 (0.80, 1.14) | 0.60 (0.49, 0.75) | 0.64 (0.51, 0.81) |
|  | [35,50) | 1.64 (1.44, 1.87) | 1.59 (1.40, 1.81) | 1.28 (1.11, 1.47) | 0.86 (0.74, 1.00) |
|  | [50,65) | 2.56 (2.29, 2.87) | 2.54 (2.26, 2.85) | 1.92 (1.69, 2.19) | 1.04 (0.90, 1.20) |
|  | [65,80) | 4.08 (3.63, 4.60) | 4.15 (3.68, 4.69) | 3.21 (2.81, 3.68) | 1.47 (1.26, 1.71) |
|  | [80,100) | 6.14 (5.23, 7.22) | 6.49 (5.51, 7.64) | 5.23 (4.36, 6.28) | 2.37 (1.94, 2.90) |
| **Male Sex** | | 1.36 (1.26, 1.46) | 1.32 (1.22, 1.42) | 1.30 (1.19, 1.42) | 1.29 (1.17, 1.41) |
| **BMI** | | 1.03 (1.03, 1.04) | 1.02 (1.02, 1.03) | 1.02 (1.01, 1.03) | 1.01 (1.00, 1.02) |
| **BMI Range**  REF: [18.5,25) | <18.5 | 1.78 (1.29, 2.45) | 1.96 (1.41, 2.73) | 1.89 (1.31, 2.73) | 1.77 (1.20, 2.59) |
|  | [25,30) | 1.63 (1.45, 1.83) | 1.18 (1.04, 1.33) | 1.12 (0.98, 1.28) | 1.11 (0.96, 1.28) |
|  | $\boldsymbol{\geq}$30 | 2.02 (1.82, 2.25) | 1.45 (1.30, 1.61) | 1.40 (1.24, 1.58) | 1.21 (1.06, 1.38) |
| **Ever-Smoker** | | 1.79 (1.65, 1.94) | 1.22 (1.12, 1.33) | 1.23 (1.12, 1.36) | 1.16 (1.05, 1.28) |
| **Smoking Status**  REF: Never-Smoker | Past-Smoker | 1.94 (1.79, 2.11) | 1.29 (1.17, 1.41) | 0.85 (0.69, 1.04) | 1.23 (1.11, 1.36) |
|  | Current-Smoker | 1.12 (0.94, 1.35) | 0.94 (0.78, 1.13) | 0.80 (0.72, 0.88) | 0.85 (0.68, 1.05) |
| **Alcohol Consumption** | | 0.84 (0.77, 0.93) | 0.81 (0.74, 0.90) | 0.80 (0.72, 0.88) | 0.86 (0.77, 0.95) |
| **Race/Ethnicity**  REF: White | Black | 1.98 (1.79, 2.19) | 2.15 (1.94, 2.39) | 1.76 (1.54, 2.01) | 1.45 (1.25, 1.68) |
|  | Other / Known Ethnicity | 1.15 (1.01, 1.30) | 1.41 (1.24, 1.61) | 1.35 (1.16, 1.56) | 1.28 (1.09, 1.50) |
|  | Other / Unknown Ethnicity | 0.57 (0.48, 0.68) | 0.56 (0.46, 0.67) | 0.55 (0.44, 0.69) | 0.50 (0.38, 0.67) |
| **SES** | Population density (1000-people/mi^2^) | 1.07 (1.05, 1.09) | 1.06 (1.04, 1.08) | 1.04 (1.02, 1.06) | 1.04 (1.01, 1.06) |
|  | NDI | 16.2 (10.9, 24.0) | 7.08 (4.35, 11.5) | 7.08 (4.35, 11.5) | 3.62 (2.09, 6.27) |
| **Comorbidity Score** | | 1.62 (1.58, 1.67) | 1.48 (1.43, 1.52) | 1.49 (1.44, 1.54) | NA |
| **Comorbidities** | Respiratory | 1.41 (1.29, 1.55) | 1.37 (1.24, 1.51) | 1.46 (1.31, 1.62) | NA |
|  | Circulatory | 3.37 (3.03, 3.74) | 2.28 (2.04, 2.55) | 2.25 (1.99, 2.54) | NA |
|  | Any Cancer | 2.56 (2.34, 2.79) | 1.79 (1.63, 1.97) | 1.77 (1.60, 1.96) | NA |
|  | Type 2 Diabetes | 3.15 (2.86, 3.46) | 2.01 (1.81, 2.23) | 2.06 (1.85, 2.29) | NA |
|  | Kidney | 5.76 (5.23, 6.36) | 3.93 (3.54, 4.37) | 3.97 (3.56, 4.44) | NA |
|  | Liver | 2.59 (2.28, 2.95) | 2.00 (1.75, 2.29) | 2.00 (1.74, 2.30) | NA |
|  | Autoimmune | 1.78 (1.61, 1.98) | 1.48 (1.33, 1.65) | 1.52 (1.36, 1.71) | NA |
| **ICU (1) vs Not (0)** | | **Unadjusted**  **(n_0_=25239, n_1_=844)** | **Adjustment 1**  **(n_0_=25237, n_1_=844)** | **Adjustment 2**  **(n_0_=19901, n_1_=660)** | **Adjustment 3**  **(n_0_=19030, n_1_=534)** |
| **Age (unit: 10-year)** | | 1.33 (1.29, 1.38) | 1.34 (1.29, 1.38) | 1.34 (1.29, 1.40) | 1.18 (1.12, 1.24) |
| **Age Range**  REF: [18,35) | [0,18) | 1.93 (1.39, 2.68) | 1.79 (1.29, 2.48) | 1.48 (1.01, 2.19) | 1.81 (1.19, 2.76) |
|  | [35,50) | 2.04 (1.55, 2.68) | 1.99 (1.51, 2.62) | 1.63 (1.19, 2.24) | 1.21 (0.84, 1.75) |
|  | [50,65) | 4.29 (3.40, 5.42) | 4.11 (3.25, 5.19) | 3.46 (2.63, 4.54) | 2.15 (1.57, 2.96) |
|  | [65,80) | 6.49 (5.11, 8.23) | 6.33 (4.98, 8.05) | 5.91 (4.49, 7.79) | 3.14 (2.26, 4.36) |
|  | [80,100) | 5.34 (3.84, 7.43) | 5.36 (3.85, 7.47) | 5.02 (3.45, 7.29) | 2.77 (1.80, 4.24) |
| **Male Sex** | | 2.02 (1.75, 2.32) | 1.96 (1.70, 2.26) | 2.01 (1.72, 2.36) | 1.95 (1.63, 2.33) |
| **BMI** | | 1.03 (1.03, 1.04) | 1.03 (1.02, 1.04) | 1.02 (1.01, 1.03) | 1.00 (0.99, 1.02) |
| **BMI Range**  REF: [18.5,25) | <18.5 | 2.14 (1.25, 3.66) | 2.35 (1.35, 4.09) | 2.30 (1.25, 4.24) | 3.06 (1.67, 5.60) |
|  | [25,30) | 1.62 (1.31, 2.01) | 1.06 (0.85, 1.33) | 0.89 (0.69, 1.14) | 0.91 (0.69, 1.19) |
|  | $\boldsymbol{\geq}$30 | 2.12 (1.74, 2.58) | 1.41 (1.16, 1.73) | 1.21 (0.97, 1.51) | 1.04 (0.81, 1.33) |
| **Ever-Smoker** | | 1.97 (1.70, 2.28) | 1.31 (1.12, 1.54) | 1.26 (1.06, 1.50) | 1.24 (1.03, 1.50) |
| **Smoking Status**  REF: Never-Smoker | Past-Smoker | 2.18 (1.87, 2.54) | 1.43 (1.21, 1.68) | 1.39 (1.16, 1.67) | 1.35 (1.12, 1.64) |
|  | Current-Smoker | 1.01 (0.70, 1.47) | 0.80 (0.55, 1.17) | 0.68 (0.44, 1.05) | 0.71 (0.45, 1.13) |
| **Alcohol Consumption** | | 0.91 (0.76, 1.10) | 0.86 (0.71, 1.04) | 0.81 (0.67, 0.99) | 0.86 (0.70, 1.05) |
| **Race/Ethnicity**  REF: White | Black | 2.34 (1.97, 2.77) | 2.54 (2.14, 3.02) | 1.77 (1.40, 2.23) | 1.37 (1.05, 1.79) |
|  | Other / Known Ethnicity | 1.05 (0.82, 1.34) | 1.28 (1.00, 1.63) | 1.27 (0.96, 1.68) | 1.27 (0.93, 1.72) |
|  | Other / Unknown Ethnicity | 1.12 (0.86, 1.46) | 1.12 (0.86, 1.46) | 1.06 (0.76, 1.47) | 0.76 (0.47, 1.22) |
| **SES** | Population density (1000-people/mi^2^) | 1.12 (1.09, 1.15) | 1.12 (1.09, 1.15) | 1.08 (1.05, 1.12) | 1.08 (1.04, 1.12) |
|  | NDI | 59.4 (31.9, 111) | 26.5 (12.2, 57.7) | 26.5 (12.2, 57.7) | 8.80 (3.41, 22.7) |
| **Comorbidity Score** | | 1.63 (1.55, 1.71) | 1.48 (1.40, 1.57) | 1.47 (1.39, 1.56) | NA |
| **Comorbidities** | Respiratory | 1.83 (1.50, 2.23) | 1.81 (1.48, 2.21) | 1.91 (1.53, 2.38) | NA |
|  | Circulatory | 4.64 (3.66, 5.88) | 3.09 (2.40, 3.98) | 2.84 (2.17, 3.73) | NA |
|  | Any Cancer | 2.17 (1.83, 2.58) | 1.44 (1.20, 1.73) | 1.50 (1.24, 1.82) | NA |
|  | Type 2 Diabetes | 3.24 (2.73, 3.85) | 1.97 (1.63, 2.37) | 1.90 (1.56, 2.31) | NA |
|  | Kidney | 6.30 (5.32, 7.46) | 4.13 (3.43, 4.98) | 3.94 (3.25, 4.79) | NA |
|  | Liver | 2.38 (1.87, 3.02) | 1.78 (1.40, 2.27) | 1.69 (1.30, 2.18) | NA |
|  | Autoimmune | 1.54 (1.26, 1.89) | 1.36 (1.10, 1.68) | 1.35 (1.08, 1.68) | NA |
| **Deceased (1) vs Alive (0)** | | **Unadjusted**  **(n_0_=25804, n_1_=485)** | **Adjustment 1**  **(n_0_=25802, n_1_=485)** | **Adjustment 2**  **(n_0_=20349, n_1_=378)** | **Adjustment 3**  **(n_0_=19391, n_1_=332)** |
| **Age (unit: 10-year)** | | 2.03 (1.91, 2.15) | 2.04 (1.92, 2.16) | 2.10 (1.95, 2.25) | 1.97 (1.82, 2.14) |
| **Age Range**  REF: [18,35) | [0,18) | 0.76 (0.19, 3.01) | 0.71 (0.18, 2.82) | 0.44 (0.08, 2.47) | 0.52 (0.09, 3.00) |
|  | [35,50) | 7.23 (3.67, 14.2) | 7.23 (3.68, 14.2) | 5.22 (2.46, 11.1) | 3.86 (1.73, 8.62) |
|  | [50,65) | 17.5 (9.32, 32.9) | 16.9 (8.99, 31.7) | 11.4 (5.62, 23.0) | 6.06 (2.83, 13.0) |
|  | [65,80) | 39.7 (21.3, 74.2) | 38.5 (20.6, 71.9) | 31.2 (15.6, 62.4) | 15.2 (7.20, 32.3) |
|  | [80,100) | 120 (64.0, 226) | 120 (63.8, 225) | 95.7 (47.5, 193) | 51.7 (24.3, 110) |
| **Male Sex** | | 2.02 (1.68, 2.43) | 1.84 (1.52, 2.22) | 2.02 (1.63, 2.50) | 1.94 (1.54, 2.43) |
| **BMI** | | 1.01 (1.00, 1.02) | 1.00 (0.99, 1.02) | 1.00 (0.99, 1.02) | 0.99 (0.97, 1.01) |
| **BMI Range**  REF: [18.5,25) | <18.5 | 2.78 (1.55, 4.99) | 3.27 (1.73, 6.20) | 2.58 (1.20, 5.53) | 3.88 (1.83, 8.25) |
|  | [25,30) | 1.58 (1.21, 2.06) | 0.92 (0.70, 1.21) | 0.80 (0.59, 1.09) | 0.76 (0.55, 1.06) |
|  | >=30 | 1.59 (1.24, 2.04) | 1.08 (0.83, 1.40) | 0.98 (0.74, 1.31) | 0.85 (0.62, 1.16) |
| **Ever-Smoker** | | 3.40 (2.78, 4.16) | 1.56 (1.27, 1.93) | 1.70 (1.34, 2.14) | 1.45 (1.14, 1.84) |
| **Smoking Status**  REF: Never-Smoker | Past-Smoker | 3.81 (3.10, 4.68) | 1.58 (1.27, 1.95) | 1.78 (1.41, 2.26) | 1.52 (1.19, 1.93) |
|  | Current-Smoker | 1.51 (0.93, 2.45) | 1.49 (0.91, 2.44) | 0.96 (0.49, 1.87) | 0.82 (0.41, 1.67) |
| **Alcohol Consumption** | | 0.57 (0.45, 0.71) | 0.57 (0.45, 0.72) | 0.54 (0.42, 0.70) | 0.62 (0.48, 0.79) |
| **Race/Ethnicity**  REF: White | Black | 1.49 (1.16, 1.91) | 1.85 (1.43, 2.40) | 1.42 (1.01, 2.01) | 1.16 (0.80, 1.70) |
|  | Other / Known Ethnicity | 0.85 (0.61, 1.19) | 1.35 (0.95, 1.91) | 1.25 (0.83, 1.87) | 1.27 (0.83, 1.94) |
|  | Other / Unknown Ethnicity | 1.50 (1.11, 2.01) | 1.41 (1.04, 1.92) | 1.10 (0.75, 1.61) | 1.16 (0.73, 1.84) |
| **SES** | Population density (1000-people/mi^2^) | 1.05 (1.01, 1.09) | 1.07 (1.03, 1.12) | 1.04 (0.99, 1.10) | 1.02 (0.96, 1.08) |
|  | NDI | 10.3 (4.13, 25.8) | 9.16 (2.94, 28.6) | 9.16 (2.94, 28.6) | 6.15 (1.65, 22.8) |
| **Comorbidity Score** | | 1.87 (1.76, 1.99) | 1.44 (1.34, 1.54) | 1.44 (1.34, 1.56) | NA |
| **Comorbidities** | Respiratory | 1.61 (1.26, 2.06) | 1.62 (1.26, 2.08) | 1.70 (1.30, 2.23) | NA |
|  | Circulatory | 7.08 (4.96, 10.1) | 2.11 (1.46, 3.05) | 2.11 (1.42, 3.14) | NA |
|  | Any Cancer | 4.32 (3.52, 5.30) | 1.64 (1.32, 2.04) | 1.64 (1.31, 2.07) | NA |
|  | Type 2 Diabetes | 5.12 (4.17, 6.30) | 2.00 (1.61, 2.49) | 2.08 (1.66, 2.62) | NA |
|  | Kidney | 7.85 (6.38, 9.66) | 2.91 (2.33, 3.64) | 2.81 (2.22, 3.56) | NA |
|  | Liver | 2.44 (1.82, 3.28) | 1.72 (1.27, 2.32) | 1.64 (1.19, 2.26) | NA |
|  | Autoimmune | 2.10 (1.65, 2.66) | 1.67 (1.30, 2.13) | 1.69 (1.31, 2.20) | NA |

Abbreviations: OR, odds ratio; ICU, intensive care unit; BMI, body mass index; NA, not applicable; REF, reference group; SES, social economics status; NDI, 2010 Neighborhood Socioeconomic Disadvantage Index; adjustment 0, unadjusted; adjustment 1, age+sex+race/ethnicity+(persons per mile^2^ in susceptibility model only); adjustment 2, adjustment 1+NDI; adjustment 3, adjustment 2+comorbidity score.

The model used was: $logit P\left( Y_{\mathrm{COVID}}=1|X, adjustment \right)=\beta_{0}+\beta_{X}X+\beta_{\mathrm{adjust}}\mathrm{adjustment}_{j}$. Here $Y_{\mathrm{COVID}}$ is various COVID-19 related outcomes under consideration (i.e., COVID-19 positive, hospitalization and ICU admission); $X$ is the variable/risk factor of interest; and $\mathrm{adjustment}_{j}$, j = 0, …,3 are the four nested covariate adjustment models listed in Supplementary Table 1.

**Supplementary Table S3. Characteristics of the COVID-19 Tested or Diagnosed Cohort, Stratified by Time Periods**

| S3A. Time Period 1 (10 March 2020 to 30 June 2020) | | | | | | | |
| --- | --- | --- | --- | --- | --- | --- | --- |
|  |  |  | **Positive Results** | | | |  |
|  | **Overall** | **Negative Results** | **Overall** | **Hospitalized** | **ICU** | **Deceased** | **Comparison Group** |
| Variable | (n = 36420) | (n = 34601) | (n = 1819) | (n = 636) | (n = 297) | (n = 129) | (n = 26363) |
| Age, y |  |  |  |  |  |  |  |
| Mean (SD) | 49.4 (22.5) | 49.2 (22.7) | 52.3 (19.1) | 58.2 (18.2) | 58.9 (16.9) | 69.0 (14.4) | 43.1 (24.6) |
| Median (IQR) | 52 (35) | 52 (35) | 53 (29) | 60 (24) | 62 (20) | 71 (22) | 43 (42) |
| <18 | 3499 (9.6) | 3460 (10) | 39 (2.1) | 13 (2) | 6 (2) | 0 (0) | 4925 (18.7) |
| [18,35) | 6684 (18.4) | 6344 (18.3) | 340 (18.7) | 64 (10.1) | 26 (8.8) | 3 (2.3) | 5994 (22.7) |
| [35,50) | 6415 (17.6) | 6026 (17.4) | 389 (21.4) | 107 (16.8) | 40 (13.5) | 10 (7.8) | 4053 (15.4) |
| [50,65) | 8850 (24.3) | 8326 (24.1) | 524 (28.8) | 194 (30.5) | 98 (33) | 33 (25.6) | 5012 (19) |
| [65,80) | 8419 (23.1) | 8035 (23.2) | 384 (21.1) | 183 (28.8) | 101 (34) | 42 (32.6) | 4565 (17.3) |
| $\boldsymbol{\geq}$80 | 2553 (7) | 2410 (7) | 143 (7.9) | 75 (11.8) | 26 (8.8) | 41 (31.8) | 1803 (6.8) |
| Gender | 15719 (43.2) | 14895 (43) | 824 (45.3) | 353 (55.5) | 187 (63) | 84 (65.1) | 12285 (46.6) |
| Primary Care at MM | 17429 (47.9) | 16633 (48.1) | 796 (43.8) | 262 (41.2) | 102 (34.3) | 42 (32.6) | 3240 (12.3) |
| BMI |  |  |  |  |  |  |  |
| Mean (SD) | 29.5 (7.7) | 29.4 (7.6) | 31.6 (8.8) | 32.4 (10.1) | 33.5 (12) | 31.3 (7.7) | 28.4 (7.4) |
| <18.5 | 546 (1.8) | 529 (1.8) | 17 (1.1) | 9 (1.5) | 1 (0.3) | 3 (2.4) | 314 (2.3) |
| [18.5,25) | 8311 (27.5) | 8010 (28) | 301 (19.2) | 95 (15.5) | 48 (16.7) | 20 (15.9) | 4597 (33.2) |
| [25,30) | 9339 (30.9) | 8893 (31.1) | 446 (28.5) | 179 (29.3) | 75 (26.1) | 38 (30.2) | 4285 (31) |
| $\boldsymbol{\geq}$30 | 12004 (39.7) | 11202 (39.1) | 802 (51.2) | 328 (53.7) | 163 (56.8) | 65 (51.6) | 4643 (33.6) |
| Smoking Status |  |  |  |  |  |  |  |
| Never | 19992 (59) | 18986 (58.7) | 1006 (64.3) | 336 (60) | 130 (56) | 38 (40.9) | 13139 (69.1) |
| Past | 10488 (30.9) | 10018 (31) | 470 (30.1) | 201 (35.9) | 95 (40.9) | 52 (55.9) | 3917 (20.6) |
| Current | 3426 (10.1) | 3338 (10.3) | 88 (5.6) | 23 (4.1) | 7 (3) | 3 (3.2) | 1954 (10.3) |
| Ever | 13914 (41) | 13356 (41.3) | 558 (35.7) | 224 (40) | 102 (44) | 55 (59.1) | 5871 (30.9) |
| Alcohol consumption | 17833 (68.4) | 17051 (68.5) | 782 (65.7) | 238 (63.3) | 108 (69.7) | 40 (58.8) | 7144 (53.7) |
| Race/ethnicity |  |  |  |  |  |  |  |
| White | 26829 (73.7) | 25929 (74.9) | 900 (49.5) | 279 (43.9) | 129 (43.4) | 68 (52.7) | 16271 (61.7) |
| Black | 4339 (11.9) | 3762 (10.9) | 577 (31.7) | 251 (39.5) | 119 (40.1) | 40 (31) | 1927 (7.3) |
| Other^b^ | 2817 (7.7) | 2650 (7.7) | 167 (9.2) | 59 (9.3) | 16 (5.4) | 6 (4.7) | 2444 (9.3) |
| Unknown^c^ | 2435 (6.7) | 2260 (6.5) | 175 (9.6) | 47 (7.4) | 33 (11.1) | 15 (11.6) | 5721 (21.7) |
| NDI, mean (SD) | 0.11 (0.09) | 0.1 (0.09) | 0.15 (0.12) | 0.18 (0.14) | 0.2 (0.15) | 0.18 (0.15) | 0.11 (0.09) |
| Population density, persons/square mile | 2268.6 (2302) | 2223.7 (2284.7) | 3092 (2458.1) | 3611.2 (2617.2) | 3763.8 (2685.4) | 3744.7 (2676) | 2348.5 (2517) |
| Comorbidity score, mean (SD) | 2.3 (1.5) | 2.3 (1.5) | 2.3 (1.6) | 3 (1.7) | 3.1 (1.8) | 3.9 (1.6) | 1.2 (1.2) |

| S3B. Time Period 2 (1 July 2020 to 31 December 2020) | | | | | | | |
| --- | --- | --- | --- | --- | --- | --- | --- |
|  |  |  | **Positive Results** | | | |  |
|  | **Overall** | **Negative Results** | **Overall** | **Hospitalized** | **ICU** | **Deceased** | **Comparison Group** |
| Variable | (n = 153838) | (n = 141290) | (n = 12548) | (n = 1357) | (n = 318) | (n = 222) | (n = 26363) |
| Age, y |  |  |  |  |  |  |  |
| Mean (SD) | 43.6 (22.8) | 43.6 (22.9) | 43.0 (21.7) | 54.2 (21.7) | 56.8 (20.8) | 70.5 (14.9) | 43.1 (24.6) |
| Median (IQR) | 43 (39) | 43 (39) | 42 (37) | 59 (35) | 61 (24.8) | 72 (21.8) | 43 (42) |
| <18 | 18528 (12) | 17488 (12.4) | 1040 (8.3) | 63 (4.6) | 18 (5.7) | 0 (0) | 4925 (18.7) |
| [18,35) | 43122 (28) | 38972 (27.6) | 4150 (33.1) | 255 (18.8) | 35 (11) | 5 (2.3) | 5994 (22.7) |
| [35,50) | 25013 (16.3) | 22852 (16.2) | 2161 (17.2) | 175 (12.9) | 35 (11) | 15 (6.8) | 4053 (15.4) |
| [50,65) | 32543 (21.2) | 29749 (21.1) | 2794 (22.3) | 360 (26.5) | 103 (32.4) | 55 (24.8) | 5012 (19) |
| [65,80) | 27383 (17.8) | 25554 (18.1) | 1829 (14.6) | 353 (26) | 98 (30.8) | 77 (34.7) | 4565 (17.3) |
| $\boldsymbol{\geq}$80 | 7249 (4.7) | 6675 (4.7) | 574 (4.6) | 151 (11.1) | 29 (9.1) | 70 (31.5) | 1803 (6.8) |
| Gender | 66693 (43.4) | 61096 (43.2) | 5597 (44.6) | 674 (49.7) | 193 (60.7) | 136 (61.3) | 12285 (46.6) |
| Primary Care at MM | 60447 (39.3) | 55437 (39.2) | 5010 (39.9) | 635 (46.8) | 113 (35.5) | 70 (31.5) | 3240 (12.3) |
| BMI |  |  |  |  |  |  |  |
| Mean (SD) | 28.7 (7.2) | 28.7 (7.2) | 29.4 (7.3) | 30.6 (7.6) | 30.7 (7.9) | 29.9 (7) | 28.4 (7.4) |
| <18.5 | 2172 (1.9) | 2032 (1.9) | 140 (1.4) | 24 (1.9) | 11 (3.7) | 5 (2.4) | 2172 (1.9) |
| [18.5,25) | 36973 (31.9) | 34170 (32.1) | 2803 (29) | 264 (20.7) | 53 (17.8) | 46 (22.2) | 36973 (31.9) |
| [25,30) | 35631 (30.7) | 32764 (30.8) | 2867 (29.6) | 396 (31.1) | 100 (33.7) | 66 (31.9) | 35631 (30.7) |
| $\boldsymbol{\geq}$30 | 41250 (35.6) | 37389 (35.2) | 3861 (39.9) | 590 (46.3) | 133 (44.8) | 90 (43.5) | 41250 (35.6) |
| Smoking Status |  |  |  |  |  |  |  |
| Never | 91299 (66.2) | 83552 (65.9) | 7747 (69.4) | 739 (57) | 149 (51.4) | 75 (39.7) | 13139 (69.1) |
| Past | 35220 (25.5) | 32317 (25.5) | 2903 (26) | 513 (39.6) | 132 (45.5) | 110 (58.2) | 3917 (20.6) |
| Current | 11374 (8.2) | 10868 (8.6) | 506 (4.5) | 45 (3.5) | 9 (3.1) | 4 (2.1) | 1954 (10.3) |
| Ever | 46594 (33.8) | 43185 (34.1) | 3409 (30.6) | 558 (43) | 141 (48.6) | 114 (60.3) | 46594 (33.8) |
| Alcohol consumption | 70899 (69.3) | 65263 (69.6) | 5636 (66) | 620 (62.5) | 120 (57.4) | 76 (50.3) | 7144 (53.7) |
| Race/ethnicity |  |  |  |  |  |  |  |
| White | 112912 (73.4) | 103777 (73.4) | 9135 (72.8) | 993 (73.2) | 236 (74.2) | 161 (72.5) | 112912 (73.4) |
| Black | 12274 (8) | 11138 (7.9) | 1136 (9.1) | 166 (12.2) | 29 (9.1) | 18 (8.1) | 12274 (8) |
| Other^b^ | 15209 (9.9) | 13996 (9.9) | 1213 (9.7) | 149 (11) | 36 (11.3) | 22 (9.9) | 15209 (9.9) |
| Unknown^c^ | 13443 (8.7) | 12379 (8.8) | 1064 (8.5) | 49 (3.6) | 17 (5.3) | 21 (9.5) | 13443 (8.7) |
| NDI, mean (SD) | 0.09 (0.08) | 0.09 (0.08) | 0.1 (0.08) | 0.11 (0.09) | 0.11 (0.08) | 0.1 (0.08) | 0.09 (0.08) |
| Population density, persons/square mile | 2234.5 (2382.3) | 2249.4  (2396.7) | 2068.1 (2207.1) | 2290 (2380.1) | 2642.8 (2868.1) | 2038.4 (2308.7) | 2234.5  (2382.3) |
| Comorbidity score, mean (SD) | 1.9 (1.4) | 1.9 (1.4) | 1.9 (1.4) | 2.9 (1.7) | 3.2 (1.5) | 3.4 (1.6) | 1.2 (1.2) |

| S3C. Time Period 3 (1 January 2021 to 3 May 2021) | | | | | | | |
| --- | --- | --- | --- | --- | --- | --- | --- |
|  |  |  | **Positive Results** | | | |  |
|  | **Overall** | **Negative Results** | **Overall** | **Hospitalized** | **ICU** | **Deceased** | **Comparison Group** |
| Variable | (n = 54681) | (n = 46870) | (n = 7811) | (n = 1073) | (n = 228) | (n = 68) | (n = 26363) |
| Age, y |  |  |  |  |  |  |  |
| Mean (SD) | 42.8 (24.0) | 43.4 (24.3) | 39.1 (21.6) | 49.0 (21.8) | 46.0 (22.4) | 67.5 (14.8) | 43.1 (24.6) |
| Median (IQR) | 44 (41) | 45 (41) | 38 (36) | 52 (34) | 52 (34) | 70 (22) | 43 (42) |
| <18 | 9849 (18) | 8514 (18.2) | 1335 (17.1) | 90 (8.4) | 34 (14.9) | 0 (0) | 4925 (18.7) |
| [18,35) | 11844 (21.7) | 9630 (20.5) | 2214 (28.3) | 213 (19.9) | 35 (15.4) | 2 (2.9) | 5994 (22.7) |
| [35,50) | 8930 (16.3) | 7395 (15.8) | 1535 (19.7) | 191 (17.8) | 35 (15.4) | 7 (10.3) | 4053 (15.4) |
| [50,65) | 11755 (21.5) | 10085 (21.5) | 1670 (21.4) | 292 (27.2) | 73 (32) | 18 (26.5) | 5012 (19) |
| [65,80) | 9652 (17.7) | 8824 (18.8) | 828 (10.6) | 217 (20.2) | 47 (20.6) | 28 (41.2) | 4565 (17.3) |
| $\boldsymbol{\geq}$80 | 2651 (4.8) | 2422 (5.2) | 229 (2.9) | 70 (6.5) | 4 (1.8) | 13 (19.1) | 1803 (6.8) |
| Gender | 24543 (44.9) | 21095 (45) | 3448 (44.1) | 522 (48.6) | 131 (57.5) | 39 (57.4) | 12285 (46.6) |
| Primary Care at MM | 19119 (35) | 15844 (33.8) | 3275 (41.9) | 461 (43) | 78 (34.2) | 19 (27.9) | 3240 (12.3) |
| BMI |  |  |  |  |  |  |  |
| Mean (SD) | 29.3 (7.4) | 29.2 (7.3) | 30.2 (7.9) | 32 (8.8) | 32.4 (8.9) | 30.7 (7.3) | 28.4 (7.4) |
| <18.5 | 730 (1.8) | 643 (1.9) | 87 (1.5) | 15 (1.6) | 3 (1.6) | 1 (1.7) | 314 (2.3) |
| [18.5,25) | 11308 (28.5) | 9817 (28.8) | 1491 (26.5) | 177 (18.4) | 35 (18.1) | 12 (20) | 4597 (33.2) |
| [25,30) | 12415 (31.3) | 10817 (31.7) | 1598 (28.4) | 267 (27.7) | 47 (24.4) | 13 (21.7) | 4285 (31) |
| $\boldsymbol{\geq}$30 | 15259 (38.4) | 12811 (37.6) | 2448 (43.5) | 504 (52.3) | 108 (56) | 34 (56.7) | 4643 (33.6) |
| Smoking Status |  |  |  |  |  |  |  |
| Never | 32908 (66.4) | 27930 (65.7) | 4978 (70.8) | 611 (59.7) | 122 (58.1) | 22 (36.7) | 13139 (69.1) |
| Past | 12283 (24.8) | 10685 (25.1) | 1598 (22.7) | 340 (33.2) | 74 (35.2) | 31 (51.7) | 3917 (20.6) |
| Current | 4369 (8.8) | 3915 (9.2) | 454 (6.5) | 72 (7) | 14 (6.7) | 7 (11.7) | 1954 (10.3) |
| Ever | 16652 (33.6) | 14600 (34.3) | 2052 (29.2) | 412 (40.3) | 88 (41.9) | 38 (63.3) | 5871 (30.9) |
| Alcohol consumption | 22864 (66) | 19747 (66.9) | 3117 (60.8) | 448 (60.6) | 86 (64.7) | 24 (49) | 7144 (53.7) |
| Race/ethnicity |  |  |  |  |  |  |  |
| White | 39799 (72.8) | 34259 (73.1) | 5540 (70.9) | 735 (68.5) | 146 (64) | 41 (60.3) | 16271 (61.7) |
| Black | 5490 (10) | 4519 (9.6) | 971 (12.4) | 186 (17.3) | 45 (19.7) | 11 (16.2) | 1927 (7.3) |
| Other^b^ | 5259 (9.6) | 4492 (9.6) | 767 (9.8) | 115 (10.7) | 24 (10.5) | 6 (8.8) | 2444 (9.3) |
| Unknown^c^ | 4133 (7.6) | 3600 (7.7) | 533 (6.8) | 37 (3.4) | 13 (5.7) | 10 (14.7) | 5721 (21.7) |
| NDI, mean (SD) | 0.1 (0.08) | 0.1 (0.08) | 0.1 (0.09) | 0.12 (0.1) | 0.13 (0.1) | 0.09 (0.06) | 0.11 (0.09) |
| Population density, persons/square mile | 2164.3 (2240.1) | 2173.9  (2234.3) | 2109.5 (2272.2) | 2308.1 (2168.2) | 2419.2 (2241.1) | 1790.1 (1618.8) | 2348.5  (2517) |
| Comorbidity score, mean (SD) | 1.8 (1.4) | 1.8 (1.4) | 1.9 (1.4) | 2.8 (1.7) | 3 (1.5) | 3.6 (1.5) | 1.2 (1.2) |
| Abbreviations: BMI, body mass index (calculated as weight in kilograms divided by height in meters squared); COVID-19, coronavirus disease 2019; ICU, intensive care unit; IQR, interquartile range; NDI, 2010 Neighborhood Socioeconomic Disadvantage Index; MM, Michigan Medicine.  a Percentages are reported as fraction of column totals excluding missing entries.  b Includes White Hispanic or unknown; Black Hispanic or unknown; Asian Hispanic,non-Hispanic, or unknown; Native American Hispanic, non-Hispanic, or unknown;Pacific Islander Hispanic, non-Hispanic, or unknown; and other Hispanic, non-Hispanic, or unknown.  c Includes missing race and/or ethnicity. | | | | | | | |

**Supplementary Table S4.** Descriptive Characteristics of the COVID-19 Tested or Diagnosed Cohort Stratified by White and Black Patients

| **White Patients** | | **Total Tested for COVID-19** | | | | | | **Matched Comparison Group**  **(n=16271)** |
| --- | --- | --- | --- | --- | --- | --- | --- | --- |
|  |  | **Overall**  **(n=182475)** | **Negative**  **(n=163976)** | **Tested Positive** | | | |  |
|  |  |  |  | **Overall**  **(n=18499)** | **Hospitalized**  **(n=2012)** | **ICU**  **(n=512)** | **Deceased**  **(n=317)** |  |
| **Variable** | | **n (%)** |  |  |  |  |  |  |
| **Age**  **(in years)** | mean (SD) | 45.7 (23.2) | 46.0 (23.4) | 42.8 (21.8) | 54.2 (21.6) | 55.3 (21.5) | 70.8 (14.7) | 42.8 (24.7) |
|  | median [IQR] | 48.0 [39.0] | 49.0 [40.0] | 42.0 [38.0] | 58.0 [33.0] | 61.0 [26.0] | 73.0 [20.0] | 42.0 [42.0] |
| **Age Range** | [0,18) | 22986 (12.6) | 21166 (12.9) | 1820 (9.8) | 115 (5.7) | 39 (7.6) | 0 (0) | 3144 (19.3) |
|  | [18,35) | 41974 (23) | 36217 (22.1) | 5757 (31.1) | 337 (16.7) | 59 (11.5) | 8 (2.5) | 3706 (22.8) |
|  | [35,50) | 29247 (16) | 26011 (15.9) | 3236 (17.5) | 262 (13) | 55 (10.7) | 21 (6.6) | 2421 (14.9) |
|  | [50,65) | 40943 (22.4) | 36778 (22.4) | 4165 (22.5) | 567 (28.2) | 159 (31.1) | 70 (22.1) | 3077 (18.9) |
|  | [65,80) | 37234 (20.4) | 34501 (21) | 2733 (14.8) | 522 (25.9) | 158 (30.9) | 117 (36.9) | 2866 (17.6) |
|  | [80,100) | 10091 (5.5) | 9303 (5.7) | 788 (4.3) | 209 (10.4) | 42 (8.2) | 101 (31.9) | 1057 (6.5) |
| **Male Sex** | | 79605 (43.6) | 71499 (43.6) | 8106 (43.8) | 1045 (51.9) | 327 (63.9) | 195 (61.5) | 7629 (46.9) |
| **Primary Care at MM** | | 74555 (40.9) | 66715 (40.7) | 7840 (42.4) | 910 (45.2) | 191 (37.3) | 112 (35.3) | 2133 (13.1) |
| **BMI, mean (SD)** | | 28.9 (7.2) | 28.9 (7.1) | 29.4 (7.5) | 31.1 (8) | 31.3 (8.2) | 29.8 (7.4) | 28.5 (7.3) |
| **BMI Range** | <18.5 | 2535 (1.8) | 2308 (1.8) | 227 (1.5) | 28 (1.5) | 6 (1.3) | 8 (2.6) | 210 (2.1) |
|  | [18.5, 25) | 43931 (30.4) | 39565 (30.4) | 4366 (29.8) | 364 (19.5) | 89 (19) | 68 (22.1) | 3304 (32.4) |
|  | [25, 30) | 45265 (31.3) | 40943 (31.5) | 4322 (29.5) | 563 (30.2) | 146 (31.1) | 106 (34.5) | 3205 (31.5) |
|  | $\boldsymbol{\geq}$30 | 52952 (36.6) | 47203 (36.3) | 5749 (39.2) | 912 (48.8) | 228 (48.6) | 125 (40.7) | 3471 (34.1) |
| **Ever-Smoker** | | 62235 (36.3) | 56921 (36.9) | 5314 (31.1) | 844 (44) | 217 (46.7) | 167 (59.4) | 4653 (33.2) |
| **Smoking Status** | Never | 109255 (63.7) | 97499 (63.1) | 11756 (68.9) | 1076 (56) | 248 (53.3) | 114 (40.6) | 9347 (66.8) |
|  | Past | 47698 (27.8) | 43222 (28) | 4476 (26.2) | 754 (39.3) | 201 (43.2) | 157 (55.9) | 3146 (22.5) |
|  | Current | 14537 (8.5) | 13699 (8.9) | 838 (4.9) | 90 (4.7) | 16 (3.4) | 10 (3.6) | 1507 (10.8) |
| **Alcohol Consumption** | | 90828 (70.5) | 81964 (70.7) | 8864 (68.3) | 951 (65.9) | 219 (65.4) | 126 (56) | 5610 (56.2) |
| **SES,**  **mean (SD)** | NDI | 0.08 (0.06) | 0.08 (0.06) | 0.08 (0.06) | 0.09 (0.06) | 0.1 (0.06) | 0.09 (0.06) | 0.09 (0.06) |
|  | Population density (people/mi^2^) | 1985.5 (2201.6) | 1994.5 (2202.7) | 1904  (2190.1) | 2089.9 (2238.4) | 2371.6 (2594.3) | 2025.9 (1982) | 2063.8  (2337.5) |
| **Comorbidity Score,**  **mean (SD)** | | 2 (1.4) | 2 (1.4) | 1.9 (1.4) | 2.9 (1.7) | 3.1 (1.6) | 3.5 (1.5) | 1.3 (1.2) |

| **Black patients** | | **Total Tested for COVID-19** | | | | | | **Matched Comparison Group**  **(n=1927)** |
| --- | --- | --- | --- | --- | --- | --- | --- | --- |
|  |  | **Overall**  **(n=22524)** | **Negative**  **(n=19423)** | **Tested Positive** | | | |  |
|  |  |  |  | **Overall**  **(n=3101)** | **Hospitalized**  **(n=603)** | **ICU**  **(n=193)** | **Deceased**  **(n=78)** |  |
| **Variable** | | n (%) |  |  |  |  |  |  |
| **Age**  **(in years)** | mean (SD) | 41.4 (21.7) | 41.4 (21.9) | 41.9 (20.4) | 50.8 (19.7) | 52.3 (18.6) | 62.8 (13.6) | 36.4 (23.4) |
|  | median [IQR] | 41.0 [35.0] | 41.0 [35.0] | 42.0 [32.0] | 53.0 [29.0] | 57.0 [26.0] | 63.0 [18.0] | 33.0 [38.0] |
| **Age Range** | [0,18) | 3126 (13.9) | 2805 (14.4) | 321 (10.4) | 25 (4.1) | 11 (5.7) | 0 (0) | 511 (26.5) |
|  | [18,35) | 6065 (26.9) | 5168 (26.6) | 897 (28.9) | 112 (18.6) | 24 (12.4) | 1 (1.3) | 482 (25) |
|  | [35,50) | 4492 (19.9) | 3781 (19.5) | 711 (22.9) | 131 (21.7) | 35 (18.1) | 13 (16.7) | 323 (16.8) |
|  | [50,65) | 5036 (22.4) | 4326 (22.3) | 710 (22.9) | 175 (29) | 73 (37.8) | 28 (35.9) | 330 (17.1) |
|  | [65,80) | 3167 (14.1) | 2806 (14.4) | 361 (11.6) | 118 (19.6) | 40 (20.7) | 23 (29.5) | 219 (11.4) |
|  | [80,100) | 638 (2.8) | 537 (2.8) | 101 (3.3) | 42 (7) | 10 (5.2) | 13 (16.7) | 62 (3.2) |
| **Male Sex** | | 9114 (40.5) | 7826 (40.3) | 1288 (41.5) | 283 (46.9) | 104 (53.9) | 49 (62.8) | 911 (47.3) |
| **Primary Care at MM** | | 9958 (44.2) | 8495 (43.7) | 1463 (47.2) | 275 (45.6) | 64 (33.2) | 26 (33.3) | 335 (17.4) |
| **BMI, mean (SD)** | | 31.3 (8.6) | 31.1 (8.5) | 32.5 (9.1) | 33.3 (10.7) | 35.2 (13.4) | 32.2 (6.8) | 30.5 (8.4) |
| **BMI Range** | <18.5 | 309 (1.7) | 273 (1.8) | 36 (1.4) | 12 (2.1) | 3 (1.7) | 2 (2.6) | 19 (1.8) |
|  | [18.5, 25) | 3770 (21.2) | 3328 (21.8) | 442 (17.3) | 85 (14.8) | 19 (10.5) | 7 (9.2) | 267 (24.7) |
|  | [25, 30) | 4909 (27.6) | 4247 (27.9) | 662 (25.9) | 150 (26.1) | 41 (22.7) | 20 (26.3) | 308 (28.5) |
|  | $\boldsymbol{\geq}$30 | 8804 (49.5) | 7389 (48.5) | 1415 (55.4) | 327 (57) | 118 (65.2) | 47 (61.8) | 485 (44.9) |
| **Ever-Smoker** | | 7506 (35.8) | 6586 (36.4) | 920 (32.1) | 228 (40.6) | 69 (42.3) | 40 (65.6) | 434 (27) |
| **Smoking Status** | Never | 13445 (64.2) | 11500 (63.6) | 1945 (67.9) | 334 (59.4) | 94 (57.7) | 21 (34.4) | 1172 (73) |
|  | Past | 4818 (23) | 4127 (22.8) | 691 (24.1) | 190 (33.8) | 60 (36.8) | 37 (60.7) | 248 (15.4) |
|  | Current | 2688 (12.8) | 2459 (13.6) | 229 (8) | 38 (6.8) | 9 (5.5) | 3 (4.9) | 186 (11.6) |
| **Alcohol Consumption** | | 9028 (58.6) | 7816 (59.2) | 1212 (54.9) | 223 (54.3) | 61 (59.8) | 22 (44) | 466 (42.1) |
| **SES, mean (SD)** | NDI | 0.21 (0.13) | 0.21 (0.13) | 0.22 (0.13) | 0.24 (0.14) | 0.27 (0.14) | 0.27 (0.14) | 0.23 (0.14) |
|  | Population density (people/mi^2^) | 3439.4 (2270.7) | 3423.7 (2279.8) | 3534.2 (2213.1) | 3834.6 (2282.5) | 4355.5 (2258.8) | 4361.2 (2455.7) | 3529.6 (2581.3) |
| **Comorbidity Score,**  **mean (SD)** | | 2.1 (1.5) | 2.1 (1.5) | 2.3 (1.6) | 3.1 (1.8) | 3.3 (1.7) | 4 (1.6) | 1.4 (1.2) |

Abbreviations: MM, Michigan Medicine; ICU, intensive care unit; BMI, body mass index; MM, Michigan Medicine; SES, social economics status; NDI, 2010 Neighborhood Socioeconomic Disadvantage Index.

**Supplementary Table S5.** Missingness of the Variables in the Full Cohort, White, and Black Patients

| Full Cohort | **COVID-19 Tested** | | | | | | **Comparison Group** |
| --- | --- | --- | --- | --- | --- | --- | --- |
|  | **Overall** | **Negative** | **COVID-19 Positive** | | | |  |
|  |  |  | **Overall** | **Hospitalized** | **ICU** | **Deceased** |  |
|  | **(n = 249075)** | **(n = 222786)** | **(n = 26289)** | **(n = 3071)** | **(n = 844)** | **(n = 485)** | **(n = 26363)** |
| **Variable** | **n (%)** |  |  |  |  |  |  |
| **Age/Age Range** | 0 (0.0) | 0 (0.0) | 0 (0.0) | 0 (0.0) | 0 (0.0) | 0 (0.0) | 11 (0.0) |
| **Male Sex** | 21 (0) | 19 (0) | 2 (0) | 0 (0) | 0 (0) | 0 (0) | 19 (0.1) |
| **Primary Care at MM** | 0 (0.0) | 0 (0.0) | 0 (0.0) | 0 (0.0) | 0 (0.0) | 0 (0.0) | 0 (0.0) |
| **BMI/BMI Range** | 59831 (24) | 53686 (24.1) | 6145 (23.4) | 219 (7.1) | 66 (7.8) | 32 (6.6) | 12523 (47.5) |
| **Ever-Smoker/ Smoking Status** | 24079 (9.7) | 21157 (9.5) | 2922 (11.1) | 187 (6.1) | 111 (13.2) | 88 (18.1) | 7353 (27.9) |
| **Alcohol Consumption** | 83290 (33.4) | 74517 (33.4) | 8773 (33.4) | 960 (31.3) | 346 (41) | 175 (36.1) | 13060 (49.5) |
| **Race Ethnicity**^a^ | 11962 (4.8) | 10741 (4.8) | 1221 (4.6) | 83 (2.7) | 47 (5.6) | 36 (7.4) | 0 (0) |
| **SES (NDI/Population density)** | 49943 (20.1) | 44381 (19.9) | 5562 (21.2) | 619 (20.2) | 184 (21.8) | 107 (22.1) | 4920 (18.7) |
| **Comorbidity Score** | 29808 (12) | 26367 (11.8) | 3441 (13.1) | 552 (18) | 246 (29.1) | 112 (23.1) | 1433 (5.4) |
| White | **(n = 182475)** | **(n = 163976)** | **(n = 18499)** | **(n = 2012)** | **(n = 512)** | **(n = 317)** | **(n = 16271)** |
| **Age/Age Range** | 0 (0) | 0 (0) | 0 (0) | 0 (0) | 0 (0) | 0 (0) | 0 (0) |
| **Male Sex** | 10 (0.0) | 9 (0.0) | 1 (0) | 0 (0) | 0 (0) | 0 (0) | 2 (0.0) |
| **Primary Care at MM** | 0 (0) | 0 (0) | 0 (0) | 0 (0) | 0 (0) | 0 (0) | 0 (0) |
| **BMI/BMI Range** | 37790 (20.7) | 33955 (20.7) | 3835 (20.7) | 145 (7.2) | 43 (8.4) | 10 (3.2) | 6080 (37.4) |
| **Ever-Smoker/ Smoking Status** | 10985 (6) | 9556 (5.8) | 1429 (7.7) | 92 (4.6) | 47 (9.2) | 36 (11.4) | 2271 (14) |
| **Alcohol Consumption** | 53592 (29.4) | 48075 (29.3) | 5517 (29.8) | 568 (28.2) | 177 (34.6) | 92 (29) | 6281 (38.6) |
| **SES (NDI/Population density)** | 32522 (17.8) | 28892 (17.6) | 3630 (19.6) | 424 (21.1) | 125 (24.4) | 66 (20.8) | 2672 (16.4) |
| **Comorbidity Score** | 16537 (9.1) | 14665 (8.9) | 1872 (10.1) | 273 (13.6) | 110 (21.5) | 55 (17.4) | 471 (2.9) |
| Black | **(n = 22524)** | **(n = 19423)** | **(n = 3101)** | **(n = 603)** | **(n = 193)** | **(n = 78)** | **(n = 1927)** |
| **Age/Age Range** | 0 (0) | 0 (0) | 0 (0) | 0 (0) | 0 (0) | 0 (0) | 0 (0) |
| **Male Sex** | 0 (0) | 0 (0) | 0 (0) | 0 (0) | 0 (0) | 0 (0) | 0 (0) |
| **Primary Care at MM** | 0 (0) | 0 (0) | 0 (0) | 0 (0) | 0 (0) | 0 (0) | 0 (0) |
| **BMI/BMI Range** | 4731 (21) | 4185 (21.5) | 546 (17.6) | 29 (4.8) | 12 (6.2) | 2 (2.6) | 848 (44) |
| **Ever-Smoker/ Smoking Status** | 1573 (7) | 1337 (6.9) | 236 (7.6) | 41 (6.8) | 30 (15.5) | 17 (21.8) | 321 (16.7) |
| **Alcohol Consumption** | 7121 (31.6) | 6226 (32.1) | 895 (28.9) | 192 (31.8) | 91 (47.2) | 28 (35.9) | 821 (42.6) |
| **SES (NDI/Population density)** | 4033 (17.9) | 3563 (18.3) | 470 (15.2) | 76 (12.6) | 22 (11.4) | 11 (14.1) | 247 (12.8) |
| **Comorbidity Score** | 2410 (10.7) | 2026 (10.4) | 384 (12.4) | 141 (23.4) | 74 (38.3) | 23 (29.5) | 74 (3.8) |

Abbreviations: MM, Michigan Medicine; ICU, intensive care unit; BMI, body mass index; MM, Michigan Medicine; SES, social economics status; NDI, 2010 Neighborhood Socioeconomic Disadvantage Index.

^a^ In the main analyses unknown race and/or ethnicity were combined into the category "Unknown race/ethnicity" to retain sample size

**Supplementary Table S6. Odds Ratios of COVID-19 Outcomes from Logistic Regression, Stratified by Time Periods**

| **Tested (1) vs. Comparison Group (0)** | | | **Full Cohort**  (n_0_=20410, n_1_=192160) | **Time Period 1***  (n_0_=20410, n_1_=31674) | **Time Period 2***  (n_0_=20410, n_1_=121497) | **Time Period 3***  (n_0_=20410, n_1_=38718) |
| --- | --- | --- | --- | --- | --- | --- |
| **Variable** | | | **OR (95% CI)** |  |  |  |
| **Age (unit: 10-year)** | | | 0.96 (0.95, 0.96) | 1.02 (1.01, 1.03) | 0.95 (0.94, 0.96) | 0.94 (0.93, 0.94) |
| **Age Range**  REF: [18,35] | | [0,18] | 0.67 (0.64, 0.70) | 0.61 (0.57, 0.65) | 0.58 (0.55, 0.61) | 1.10 (1.04, 1.16) |
|  |  | [35,50] | 0.93 (0.88, 0.97) | 1.19 (1.12, 1.27) | 0.85 (0.81, 0.90) | 1.10 (1.03, 1.16) |
|  |  | [50,65] | 0.79 (0.76, 0.83) | 1.03 (0.97, 1.09) | 0.72 (0.69, 0.76) | 0.96 (0.90, 1.01) |
|  |  | [65,80] | 0.65 (0.62, 0.68) | 0.91 (0.86, 0.97) | 0.58 (0.55, 0.61) | 0.75 (0.71, 0.80) |
|  |  | [80,100] | 0.44 (0.41, 0.47) | 0.69 (0.64, 0.76) | 0.37 (0.34, 0.40) | 0.52 (0.47, 0.56) |
| **Male Sex** | | | 0.83 (0.81, 0.86) | 0.80 (0.77, 0.83) | 0.82 (0.80, 0.85) | 0.88 (0.85, 0.91) |
| **BMI** | | | 1.00 (1.00, 1.01) | 1.01 (1.00, 1.01) | 1.00 (1.00, 1.00) | 1.01 (1.00, 1.01) |
| **BMI Range**  REF: [18.5,25] | | <18.5 | 0.86 (0.75, 0.98) | 0.96 (0.81, 1.13) | 0.83 (0.73, 0.96) | 0.89 (0.76, 1.05) |
|  |  | [25,30] | 1.10 (1.05, 1.16) | 1.12 (1.06, 1.19) | 1.09 (1.04, 1.14) | 1.15 (1.09, 1.22) |
|  |  | >=30 | 1.08 (1.03, 1.14) | 1.16 (1.09, 1.23) | 1.05 (1.00, 1.10) | 1.15 (1.09, 1.22) |
| **Ever-Smoker** | | | 1.05 (1.01, 1.09) | 1.18 (1.13, 1.24) | 1.03 (0.99, 1.07) | 1.02 (0.98, 1.07) |
| **Smoking Status**  REF: Never-Smoker | | Past-Smoker | 1.15 (1.10, 1.21) | 1.27 (1.21, 1.34) | 1.11 (1.05, 1.17) | 1.11 (1.05, 1.17) |
|  |  | Current-Smoker | 0.86 (0.81, 0.91) | 1.00 (0.93, 1.07) | 0.86 (0.80, 0.92) | 0.86 (0.80, 0.92) |
| **Alcohol Consumption** | | | 1.94 (1.86, 2.02) | 1.97 (1.87, 2.07) | 2.00 (1.92, 2.09) | 1.65 (1.57, 1.73) |
| **Race/Ethnicity**  REF: White | | Black | 1.15 (1.08, 1.22) | 1.35 (1.26, 1.45) | 1.06 (0.99, 1.13) | 1.27 (1.18, 1.36) |
|  |  | Other / Known Ethnicity | 0.93 (0.88, 0.98) | 0.85 (0.80, 0.91) | 0.94 (0.89, 0.99) | 0.96 (0.90, 1.02) |
|  |  | Other / Unknown Ethnicity | 0.35 (0.33, 0.36) | 0.32 (0.30, 0.34) | 0.36 (0.34, 0.38) | 0.36 (0.34, 0.38) |
| **SES** | | Population density (1000-people/mi^2^) | 1.01 (1.00, 1.01) | 1.00 (0.99, 1.01) | 1.02 (1.01, 1.02) | 0.99 (0.98, 1.00) |
|  |  | NDI | 0.17 (0.14, 0.21) | 0.48 (0.37, 0.62) | 0.12 (0.10, 0.15) | 0.24 (0.18, 0.30) |
| **Comorbidity Score** | | | 1.62 (1.60, 1.64) | 1.72 (1.69, 1.75) | 1.58 (1.55, 1.60) | 1.63 (1.60, 1.65) |
| **Comorbidities** | | Respiratory | 2.55 (2.48, 2.63) | 2.79 (2.68, 2.90) | 2.47 (2.39, 2.54) | 2.55 (2.46, 2.64) |
|  |  | Circulatory | 2.44 (2.36, 2.52) | 2.91 (2.79, 3.03) | 2.29 (2.21, 2.36) | 2.44 (2.35, 2.54) |
|  |  | Any Cancer | 1.37 (1.31, 1.42) | 1.68 (1.60, 1.76) | 1.30 (1.25, 1.36) | 1.36 (1.30, 1.43) |
|  |  | Type 2 Diabetes | 1.77 (1.67, 1.87) | 2.04 (1.92, 2.17) | 1.67 (1.58, 1.76) | 1.75 (1.64, 1.86) |
|  |  | Kidney | 2.42 (2.25, 2.59) | 3.48 (3.23, 3.76) | 2.20 (2.05, 2.37) | 2.13 (1.97, 2.31) |
|  |  | Liver | 2.75 (2.50, 3.02) | 3.60 (3.26, 3.99) | 2.54 (2.31, 2.79) | 2.62 (2.36, 2.90) |
|  |  | Autoimmune | 2.04 (1.92, 2.16) | 2.27 (2.13, 2.42) | 1.95 (1.84, 2.07) | 2.00 (1.88, 2.13) |
| **Positive (1) vs Comparison Group (0)** | | | **Full Cohort**  (n0=26363, n1=26289) | **Time Period 1**  (n0=26363, n1=1819) | **Time Period 2**  (n0=26363, n1=12548) | **Time Period 3**  (n0=26363, n1=7811) |
| **Variable** | | | **OR (95% CI)** |  |  |  |
| **Age (unit: 10-year)** | | | 0.92 (0.91, 0.93) | 1.08 (1.05, 1.11) | 0.94 (0.93, 0.95) | 0.85 (0.84, 0.86) |
| **Age Range**  REF: [18,35) | [0,18) | | 0.48 (0.45, 0.52) | 0.15 (0.11, 0.23) | 0.37 (0.34, 0.40) | 0.89 (0.81, 0.98) |
|  | [35,50) | | 0.87 (0.81, 0.93) | 1.41 (1.17, 1.69) | 0.79 (0.73, 0.86) | 1.02 (0.93, 1.12) |
|  | [50,65) | | 0.68 (0.64, 0.73) | 1.21 (1.01, 1.45) | 0.67 (0.62, 0.72) | 0.69 (0.63, 0.76) |
|  | [65,80) | | 0.39 (0.36, 0.42) | 0.91 (0.74, 1.10) | 0.41 (0.37, 0.44) | 0.29 (0.26, 0.33) |
|  | [80,100) | | 0.28 (0.25, 0.31) | 0.80 (0.61, 1.04) | 0.33 (0.29, 0.37) | 0.20 (0.16, 0.24) |
| **Male Sex** | | | 0.85 (0.81, 0.88) | 0.82 (0.73, 0.92) | 0.86 (0.82, 0.91) | 0.85 (0.80, 0.90) |
| **BMI** | | | 1.02 (1.02, 1.02) | 1.02 (1.02, 1.03) | 1.01 (1.01, 1.02) | 1.02 (1.02, 1.03) |
| **BMI Range**  REF: [18.5,25) | <18.5 | | 0.79 (0.66, 0.96) | 0.82 (0.46, 1.46) | 0.71 (0.56, 0.90) | 0.85 (0.65, 1.13) |
|  | [25,30) | | 1.25 (1.17, 1.33) | 1.37 (1.14, 1.64) | 1.21 (1.12, 1.31) | 1.33 (1.21, 1.47) |
|  | >=30 | | 1.44 (1.35, 1.53) | 1.80 (1.53, 2.13) | 1.38 (1.28, 1.49) | 1.64 (1.49, 1.79) |
| **Ever-Smoker** | | | 0.89 (0.84, 0.94) | 0.89 (0.77, 1.01) | 0.89 (0.84, 0.95) | 0.93 (0.87, 1.01) |
| **Smoking Status**  REF: Never-Smoker | Past-Smoker | | 1.08 (1.02, 1.15) | 1.04 (0.90, 1.21) | 1.11 (1.04, 1.19) | 1.08 (0.99, 1.17) |
|  | Current-Smoker | | 0.52 (0.48, 0.57) | 0.55 (0.43, 0.71) | 0.46 (0.41, 0.51) | 0.66 (0.59, 0.75) |
| **Alcohol Consumption** | | | 1.72 (1.63, 1.82) | 1.72 (1.50, 1.98) | 1.75 (1.64, 1.87) | 1.54 (1.43, 1.67) |
| **Race/Ethnicity**  REF: White | Black | | 1.48 (1.37, 1.61) | 4.17 (3.53, 4.92) | 1.19 (1.07, 1.31) | 1.52 (1.36, 1.70) |
|  | Other / Known Ethnicity | | 0.99 (0.92, 1.06) | 1.29 (1.04, 1.60) | 0.98 (0.90, 1.07) | 0.94 (0.84, 1.04) |
|  | Other / Unknown Ethnicity | | 0.38 (0.35, 0.41) | 0.53 (0.42, 0.67) | 0.39 (0.35, 0.43) | 0.38 (0.33, 0.43) |
| **SES** | Population density (1000-people/mi^2^) | | 0.99 (0.98, 1.00) | 1.06 (1.03, 1.08) | 0.97 (0.96, 0.98) | 0.96 (0.95, 0.98) |
|  | NDI | | 0.28 (0.21, 0.37) | 1.00 (0.52, 1.94) | 0.27 (0.19, 0.39) | 0.36 (0.24, 0.55) |
| **Comorbidity Score** | | | 1.67 (1.64, 1.70) | 1.62 (1.55, 1.70) | 1.58 (1.55, 1.62) | 1.72 (1.67, 1.76) |
| **Comorbidities** | Respiratory | | 2.89 (2.77, 3.01) | 2.69 (2.38, 3.05) | 2.61 (2.48, 2.75) | 3.04 (2.85, 3.24) |
|  | Circulatory | | 2.50 (2.39, 2.61) | 2.69 (2.36, 3.08) | 2.32 (2.20, 2.46) | 2.60 (2.43, 2.77) |
|  | Any Cancer | | 1.25 (1.18, 1.32) | 1.22 (1.05, 1.40) | 1.21 (1.13, 1.29) | 1.29 (1.19, 1.40) |
|  | Type 2 Diabetes | | 2.02 (1.88, 2.16) | 2.18 (1.87, 2.55) | 1.85 (1.71, 2.01) | 2.25 (2.05, 2.47) |
|  | Kidney | | 2.62 (2.41, 2.85) | 2.86 (2.41, 3.40) | 2.47 (2.24, 2.72) | 2.73 (2.45, 3.06) |
|  | Liver | | 2.92 (2.62, 3.26) | 2.85 (2.26, 3.60) | 2.57 (2.27, 2.92) | 3.10 (2.69, 3.56) |
|  | Autoimmune | | 2.16 (2.02, 2.32) | 2.53 (2.16, 2.96) | 2.04 (1.89, 2.22) | 2.07 (1.88, 2.28) |
| **Deceased (1) vs Alive (0)** | | | **Full Cohort**  (n0=25804, n1=485) | **Time Period 1**  (n0=1690, n1=129) | **Time Period 2**  (n0=12326, n1=222) | **Time Period 3**  (n0=7743, n1=68) |
| **Variable** | | | **OR (95% CI)** |  |  |  |
| **Age (unit: 10-year)** | | | 1.98 (1.83, 2.15) | 1.78 (1.49, 2.13) | 2.01 (1.79, 2.25) | 2.01 (1.63, 2.47) |
| **Age Range**  REF: [18,35) | [0,18) | | 0.53 (0.09, 3.01) | 1.31 (0.06, 30.1) | 0.31 (0.02, 5.42) | NA (NA, NA) |
|  | [35,50) | | 3.85 (1.72, 8.62) | 1.67 (0.39, 7.22) | 2.53 (0.92, 6.97) | NA (NA, NA) |
|  | [50,65) | | 6.07 (2.84, 13.0) | 2.49 (0.65, 9.59) | 4.54 (1.83, 11.2) | NA (NA, NA) |
|  | [65,80) | | 15.2 (7.20, 32.2) | 4.69 (1.25, 17.6) | 10.7 (4.39, 26.0) | NA (NA, NA) |
|  | [80,100) | | 52.2 (24.5, 111) | 23.3 (6.08, 89.4) | 35.0 (14.3, 85.7) | NA (NA, NA) |
| **Male Sex** | | | 1.93 (1.54, 2.43) | 2.57 (1.55, 4.26) | 1.72 (1.25, 2.37) | 1.92 (1.07, 3.42) |
| **BMI** | | | 0.99 (0.97, 1.01) | 1.03 (0.99, 1.06) | 0.98 (0.96, 1.01) | 1.00 (0.95, 1.04) |
| **BMI Range**  REF: [18.5,25) | <18.5 | | 3.92 (1.84, 8.32) | 7.51 (1.08, 52.5) | 2.95 (0.89, 9.77) | 5.36 (0.84, 34.4) |
|  | [25,30) | | 0.75 (0.54, 1.05) | 1.01 (0.43, 2.39) | 0.68 (0.44, 1.06) | 0.51 (0.21, 1.26) |
|  | >=30 | | 0.84 (0.62, 1.15) | 2.03 (0.92, 4.48) | 0.65 (0.43, 1.00) | 0.95 (0.44, 2.06) |
| **Ever-Smoker** | | | 1.44 (1.13, 1.84) | 1.04 (0.61, 1.80) | 1.73 (1.23, 2.43) | 1.64 (0.89, 3.02) |
| **Smoking Status**  REF: Never-Smoker | Past-Smoker | | 1.51 (1.18, 1.93) | 1.13 (0.65, 1.95) | 1.80 (1.27, 2.54) | 1.62 (0.86, 3.04) |
|  | Current-Smoker | | 0.82 (0.40, 1.66) | 0.45 (0.08, 2.54) | 0.99 (0.33, 2.97) | 2.06 (0.65, 6.50) |
| **Alcohol Consumption** | | | 0.62 (0.48, 0.79) | 0.86 (0.49, 1.54) | 0.54 (0.38, 0.77) | 0.53 (0.28, 1.00) |
| **Race/Ethnicity**  REF: White | Black | | 1.31 (0.93, 1.85) | 0.88 (0.48, 1.60) | 1.13 (0.64, 1.99) | 1.22 (0.46, 3.28) |
|  | Other / Known Ethnicity | | 1.28 (0.83, 1.95) | 1.37 (0.53, 3.56) | 0.95 (0.51, 1.80) | 2.01 (0.79, 5.11) |
|  | Other / Unknown Ethnicity | | 1.19 (0.75, 1.89) | 1.63 (0.60, 4.48) | 1.01 (0.52, 1.94) | 1.67 (0.60, 4.64) |
| **SES** | Population density (1000-people/mi^2^) | | 2.63 (0.19, 36.7) | 18.6 (0.16, 2170) | 0.13 (0, 8.30) | 0.24 (0, 754) |
|  | NDI | | 1.02 (0.96, 1.08) | 1.08 (0.97, 1.21) | 0.98 (0.90, 1.06) | 0.98 (0.84, 1.16) |
| **Comorbidity Score** | | | 1.44 (1.34, 1.56) | 1.59 (1.35, 1.88) | 1.39 (1.25, 1.54) | 1.53 (1.26, 1.87) |
| **Comorbidities** | Respiratory | | 1.70 (1.30, 2.24) | 2.64 (1.39, 4.99) | 1.66 (1.14, 2.39) | 1.26 (0.65, 2.45) |
|  | Circulatory | | 2.11 (1.42, 3.13) | 2.49 (1.09, 5.69) | 1.57 (0.95, 2.60) | 3.82 (1.09, 13.4) |
|  | Any Cancer | | 1.65 (1.31, 2.07) | 2.45 (1.49, 4.03) | 1.52 (1.10, 2.10) | 1.51 (0.84, 2.73) |
|  | Type 2 Diabetes | | 2.08 (1.65, 2.61) | 2.41 (1.47, 3.97) | 1.88 (1.36, 2.61) | 2.71 (1.51, 4.84) |
|  | Kidney | | 2.80 (2.22, 3.55) | 4.22 (2.52, 7.06) | 2.13 (1.52, 2.98) | 4.65 (2.53, 8.54) |
|  | Liver | | 1.63 (1.18, 2.25) | 1.32 (0.61, 2.87) | 2.30 (1.50, 3.54) | 1.34 (0.59, 3.07) |
|  | Autoimmune | | 1.70 (1.31, 2.20) | 1.52 (0.87, 2.66) | 1.81 (1.25, 2.62) | 1.37 (0.69, 2.74) |

Abbreviations: OR, odds ratio; ICU, intensive care unit; BMI, body mass index; NA, not applicable; REF, reference group; SES, social economics status; NDI, 2010 Neighborhood Socioeconomic Disadvantage Index; Time Period 1, 10 March 2020 to 30 June 2020; Time Period 2, 1 July 2020 to 31 December 2020; Time Period 3, 1 January 2021 to 3 May 2021.

The model used was: $logit P\left( Y_{\mathrm{COVID}}=1|X, adjustment \right)=\beta_{0}+\beta_{X}X+\beta_{\mathrm{adjust}}\mathrm{adjustment}_{3}$. Here $Y_{\mathrm{COVID}}$ is various COVID-19 related outcomes under consideration (i.e., COVID-19 positive, hospitalization and ICU admission); $X$ is the variable/risk factor of interest; and $\mathrm{adjustment}_{3}$ is listed in Supplementary Table 1.

Note: green represents significant OR<1 (protective effect), orange represents significant OR>=1 (risk effect) under P<0.05.

**Supplementary Table S7. Proportion of Transferred Patients by Outcome and Time Periods**

| **Outcome** | **Transferred** | **Tested Positive** | | |  | **Diagnosed** | **Total** |
| --- | --- | --- | --- | --- | --- | --- | --- |
|  |  | **T1** | **T2** | **T3** |  | **Unknown Test Date** |  |
| Hospitalized | Yes | 123 | 76 | 50 |  | 0 | 249 |
|  | No | 513 | 1281 | 1023 |  | 5 | 2822 |
|  | % | 19.3% | 5.6% | 4.7% |  | 0.0% | 8.1% |
| ICU | Yes | 108 | 53 | 41 |  | 0 | 202 |
|  | No | 189 | 265 | 187 |  | 1 | 642 |
|  | % | 36.4% | 16.7% | 18.0% |  | 0.0% | 23.9% |
| Deceased | Yes | 37 | 23 | 6 |  | 0 | 66 |
|  | No | 92 | 199 | 62 |  | 66 | 419 |
|  | % | 28.7% | 10.4% | 8.8% |  | 0.0% | 13.6% |

Abbreviations: ICU, intensive care unit; T1, 10 March 2020 – 30 June 2020; T2, 1 July 2020 – 31 December 2020; T3, 1 January 2021 – 3 May 2021.

**Supplementary Table S8. Sensitivity Analysis Using Patients with Primary Care at Michigan Medicine, Stratified by Time Periods.**

| **Hospitalization (1) vs Not (0)** | | **Full Cohort**  (n0=8536, n1=1193) | **Time Period 1**  (n0=484, n1=225) | **Time Period 2**  (n0=3978, n1=566) | **Time Period 3**  (n0=2549, n1=401) |
| --- | --- | --- | --- | --- | --- |
| **Age (unit: 10-year)** | | 1.21 (1.17, 1.26) | 1.21 (1.09, 1.34) | 1.22 (1.16, 1.29) | 1.18 (1.10, 1.25) |
| **Age Range**  REF: [18,35) | [0,18) | 0.42 (0.30, 0.60) | 1.88 (0.60, 5.92) | 0.43 (0.25, 0.74) | 0.32 (0.20, 0.54) |
|  | [35,50) | 0.72 (0.58, 0.89) | 0.75 (0.42, 1.32) | 0.74 (0.54, 1.00) | 0.65 (0.46, 0.92) |
|  | [50,65) | 0.88 (0.72, 1.07) | 1.03 (0.61, 1.75) | 0.90 (0.68, 1.20) | 0.73 (0.52, 1.02) |
|  | [65,80) | 1.49 (1.20, 1.86) | 2.11 (1.16, 3.83) | 1.52 (1.11, 2.07) | 1.37 (0.92, 2.03) |
|  | [80,100) | 3.53 (2.67, 4.68) | 3.50 (1.62, 7.57) | 3.66 (2.46, 5.44) | 2.65 (1.54, 4.56) |
| **Male Sex** | | 1.31 (1.15, 1.49) | 1.72 (1.21, 2.45) | 1.23 (1.02, 1.48) | 1.28 (1.02, 1.60) |
| **BMI** | | 1.01 (1.00, 1.01) | 1.02 (0.99, 1.04) | 1.00 (0.99, 1.01) | 1.00 (0.99, 1.02) |
| **BMI Range**  REF: [18.5,25) | <18.5 | 1.67 (0.94, 2.96) | 1.84 (0.17, 19.8) | 1.97 (0.88, 4.42) | 1.57 (0.63, 3.92) |
|  | [25,30) | 1.06 (0.87, 1.28) | 1.48 (0.85, 2.55) | 1.06 (0.81, 1.39) | 0.90 (0.64, 1.26) |
|  | >=30 | 1.13 (0.95, 1.35) | 1.81 (1.08, 3.04) | 1.03 (0.80, 1.32) | 1.06 (0.78, 1.45) |
| **Ever-Smoker** | | 1.14 (1.00, 1.30) | 1.23 (0.85, 1.78) | 1.17 (0.96, 1.43) | 1.10 (0.86, 1.39) |
| **Smoking Status**  REF: Never-Smoker | Past-Smoker | 1.22 (1.06, 1.40) | 1.47 (0.99, 2.16) | 1.25 (1.02, 1.52) | 1.14 (0.88, 1.47) |
|  | Current-Smoker | 0.76 (0.56, 1.04) | 0.50 (0.22, 1.16) | 0.69 (0.40, 1.19) | 0.95 (0.61, 1.48) |
| **Alcohol Consumption** | | 0.79 (0.69, 0.91) | 0.64 (0.44, 0.95) | 0.82 (0.67, 1.01) | 0.85 (0.67, 1.09) |
| **Race/Ethnicity**  REF: White | Black | 1.46 (1.20, 1.78) | 1.25 (0.80, 1.95) | 1.09 (0.78, 1.53) | 1.61 (1.14, 2.28) |
|  | Other / Known Ethnicity | 1.27 (1.03, 1.58) | 1.70 (0.95, 3.07) | 1.27 (0.94, 1.72) | 1.01 (0.67, 1.52) |
|  | Other / Unknown Ethnicity | 0.61 (0.41, 0.92) | 1.15 (0.46, 2.85) | 0.50 (0.27, 0.90) | 0.51 (0.24, 1.07) |
| **SES** | Population density (1000-people/mi^2^) | 1.05 (1.01, 1.08) | 1.02 (0.93, 1.11) | 1.08 (1.03, 1.13) | 1.02 (0.96, 1.08) |
|  | NDI | 5.82 (2.68, 12.6) | 5.24 (0.88, 31.1) | 7.24 (2.17, 24.1) | 2.94 (0.72, 12.0) |
| **Comorbidity Score** | | 1.45 (1.38, 1.52) | 1.48 (1.30, 1.69) | 1.48 (1.38, 1.59) | 1.53 (1.40, 1.67) |
| **Comorbidities** | Respiratory | 1.05 (0.88, 1.25) | 1.19 (0.77, 1.86) | 1.16 (0.89, 1.51) | 0.97 (0.70, 1.34) |
|  | Circulatory | 1.90 (1.57, 2.30) | 2.53 (1.54, 4.15) | 1.64 (1.25, 2.15) | 2.41 (1.72, 3.39) |
|  | Any Cancer | 1.77 (1.54, 2.03) | 1.71 (1.18, 2.49) | 1.92 (1.57, 2.35) | 1.89 (1.47, 2.42) |
|  | Type 2 Diabetes | 2.06 (1.78, 2.39) | 1.93 (1.29, 2.90) | 2.31 (1.86, 2.87) | 1.92 (1.48, 2.49) |
|  | Kidney | 3.78 (3.24, 4.40) | 4.34 (2.80, 6.70) | 3.67 (2.92, 4.62) | 4.78 (3.64, 6.29) |
|  | Liver | 1.52 (1.26, 1.82) | 1.55 (0.91, 2.63) | 1.78 (1.36, 2.33) | 1.55 (1.12, 2.14) |
|  | Autoimmune | 1.49 (1.29, 1.73) | 1.76 (1.20, 2.60) | 1.50 (1.21, 1.87) | 1.58 (1.21, 2.06) |
| **ICU (1) vs Not (0)** | | **Full Cohort**  (n0=9464, n1=265) | **Time Period 1**  (n0=623, n1=86) | **Time Period 2**  (n0=4436, n1=108) | **Time Period 3**  (n0=2880, n1=70) |
| **Age (unit: 10-year)** | | 1.27 (1.18, 1.37) | 1.21 (1.04, 1.40) | 1.49 (1.35, 1.64) | 1.21 (1.09, 1.34) |
| **Age Range**  REF: [18,35) | [0,18) | 0.73 (0.35, 1.52) | 2.73 (0.55, 13.7) | 0.28 (0.05, 1.49) | 1.29 (0.53, 3.12) |
|  | [35,50) | 0.93 (0.56, 1.54) | 1.03 (0.38, 2.79) | 1.12 (0.53, 2.38) | 1.29 (0.55, 3.02) |
|  | [50,65) | 1.75 (1.13, 2.70) | 2.20 (0.93, 5.19) | 2.41 (1.31, 4.42) | 2.58 (1.22, 5.46) |
|  | [65,80) | 3.19 (2.02, 5.04) | 3.06 (1.23, 7.63) | 5.79 (3.19, 10.5) | 5.06 (2.30, 11.1) |
|  | [80,100) | 3.05 (1.70, 5.48) | 2.86 (0.96, 8.51) | 6.47 (3.07, 13.6) | 2.02 (0.49, 8.33) |
| **Male Sex** | | 2.19 (1.70, 2.81) | 2.88 (1.78, 4.66) | 2.07 (1.42, 3.02) | 1.85 (1.17, 2.91) |
| **BMI** | | 1.00 (0.98, 1.02) | 1.04 (1.00, 1.07) | 1.01 (0.98, 1.03) | 1.00 (0.97, 1.03) |
| **BMI Range**  REF: [18.5,25) | <18.5 | 2.68 (1.05, 6.80) | 0 (0, 0) | 5.00 (1.90, 13.2) | 1.33 (0.24, 7.44) |
|  | [25,30) | 0.75 (0.52, 1.09) | 1.14 (0.52, 2.50) | 1.20 (0.72, 2.00) | 0.86 (0.43, 1.70) |
|  | >=30 | 0.88 (0.63, 1.23) | 1.54 (0.74, 3.19) | 1.26 (0.78, 2.03) | 1.17 (0.64, 2.12) |
| **Ever-Smoker** | | 1.25 (0.96, 1.62) | 1.24 (0.75, 2.04) | 2.44 (1.68, 3.54) | 1.72 (1.09, 2.71) |
| **Smoking Status**  REF: Never-Smoker | Past-Smoker | 1.33 (1.02, 1.74) | 1.45 (0.87, 2.41) | 2.61 (1.79, 3.81) | 1.86 (1.15, 3.01) |
|  | Current-Smoker | 0.81 (0.44, 1.51) | 0.43 (0.11, 1.68) | 1.46 (0.55, 3.86) | 1.33 (0.54, 3.26) |
| **Alcohol Consumption** | | 0.76 (0.58, 1.00) | 1.21 (0.70, 2.07) | 0.57 (0.38, 0.85) | 0.72 (0.43, 1.19) |
| **Race/Ethnicity**  REF: White | Black | 1.49 (1.03, 2.17) | 1.12 (0.62, 2.04) | 0.76 (0.37, 1.56) | 2.24 (1.33, 3.75) |
|  | Other / Known Ethnicity | 1.38 (0.91, 2.08) | 0.77 (0.29, 2.02) | 1.49 (0.88, 2.52) | 1.31 (0.62, 2.73) |
|  | Other / Unknown Ethnicity | 0.60 (0.26, 1.43) | 0.54 (0.10, 3.04) | 1.16 (0.48, 2.78) | 0.19 (0.01, 3.15) |
| **SES** | Population density (1000-people/mi^2^) | 1.11 (1.04, 1.17) | 1.10 (0.98, 1.24) | 1.17 (1.09, 1.25) | 1.04 (0.93, 1.16) |
|  | NDI | 7.55 (1.86, 30.7) | 1.64 (0.15, 17.5) | 10.4 (1.51, 71.7) | 10.1 (1.09, 93.1) |
| **Comorbidity Score** | | 1.43 (1.30, 1.56) | 1.43 (1.20, 1.69) | 1.70 (1.51, 1.91) | 1.52 (1.32, 1.75) |
| **Comorbidities** | Respiratory | 1.44 (0.97, 2.15) | 1.58 (0.79, 3.18) | 1.95 (1.03, 3.71) | 0.97 (0.51, 1.82) |
|  | Circulatory | 1.84 (1.21, 2.81) | 2.37 (1.06, 5.29) | 3.69 (2.04, 6.66) | 2.30 (1.27, 4.17) |
|  | Any Cancer | 1.43 (1.09, 1.87) | 1.29 (0.77, 2.14) | 2.23 (1.52, 3.27) | 2.61 (1.64, 4.16) |
|  | Type 2 Diabetes | 2.12 (1.61, 2.79) | 1.76 (1.04, 2.97) | 5.05 (3.46, 7.38) | 2.26 (1.37, 3.73) |
|  | Kidney | 3.60 (2.72, 4.76) | 3.77 (2.22, 6.41) | 5.50 (3.72, 8.13) | 6.23 (3.91, 9.94) |
|  | Liver | 1.34 (0.94, 1.89) | 0.83 (0.39, 1.78) | 2.94 (1.81, 4.77) | 1.95 (1.03, 3.70) |
|  | Autoimmune | 1.56 (1.17, 2.07) | 2.25 (1.34, 3.76) | 1.63 (1.05, 2.52) | 1.60 (0.93, 2.76) |

Abbreviations: OR, odds ratio; ICU, intensive care unit; BMI, body mass index; NA, not applicable; REF, reference group; SES, social economics status; NDI, 2010 Neighborhood Socioeconomic Disadvantage Index. Time Period 1, 10 March 2020 to 30 June 2020; Time Period 2, 1 July 2020 to 31 December 2020; Time Period 3, 1 January 2021 to 3 May 2021.

Note: green represents significant OR<1 (protective effect), orange represents significant OR>=1 (risk effect) under P<0.05.
